# Multiple non-climatic drivers of food insecurity reinforce climate change maladaptation trajectories among Peruvian Indigenous Shawi in the Amazon

**DOI:** 10.1371/journal.pone.0205714

**Published:** 2018-10-16

**Authors:** Carol Zavaleta, Lea Berrang-Ford, James Ford, Alejandro Llanos-Cuentas, César Cárcamo, Nancy A. Ross, Guillermo Lancha, Mya Sherman, Sherilee L. Harper, the Indigenous Health and Adaption to Climate Change Research Group

**Affiliations:** 1 Department of Geography, McGill University, Montreal, Quebec, Canada; 2 Facultad de Salud Pública, Universidad Peruana Cayetano Heredia, Lima, Perú; 3 Shawi community, Alto Amazonas, Loreto, Perú; 4 Department of Population Medicine, University of Guelph, Ontario, Canada; University of Minnesota, UNITED STATES

## Abstract

**Background:**

Climate change is affecting food systems globally, with implications for food security, nutrition, and the health of human populations. There are limited data characterizing the current and future consequences of climate change on local food security for populations already experiencing poor nutritional indicators. Indigenous Amazonian populations have a high reported prevalence of nutritional deficiencies. This paper characterizes the food system of the Shawi of the Peruvian Amazon, climatic and non-climatic drivers of their food security vulnerability to climate change, and identifies potential maladaptation trajectories.

**Methods and findings:**

Semi-structured interviews with key informants (n = 24), three photovoice workshops (n = 17 individuals), transect walks (n = 2), a food calendar exercise, and two community dissemination meetings (n = 30 individuals), were conducted within two Shawi communities in Balsapuerto District in the Peruvian Loreto region between June and September of 2014. The Shawi food system was based on three main food sub-systems (forest, farming and externally-sourced). Shawi reported collective, gendered, and emotional notions related to their food system activities. Climatic and non-climatic drivers of food security vulnerability among Shawi participants acted at proximal and distal levels, and mutually reinforced key maladaptation trajectories, including: 1) a growing population and natural resource degradation coupled with limited opportunities to increase incomes, and 2) a desire for education and deforestation reinforced by governmental social and food interventions.

**Conclusion:**

A series of maladaptive trajectories have the potential to increase social and nutritional inequities for the Shawi. Transformational food security adaptation should include consideration of Indigenous perceptions and priorities, and should be part of Peruvian food and socioeconomic development policies.

## Introduction

Climate change is affecting food systems globally, with implications for food security, nutrition and the health of human populations[1, 2]. Despite a growing body of research characterizing climatic impacts on food systems, research has predominantly focused on the macro level (global or regional) and climate impacts on agriculture [1, 3–6]. Less is known about how human populations react to climatic impacts on food security at the local level, and how climate and weather interact with other drivers of health [7–10]. The ways in which climate change risks impact food security and human nutrition are mediated by local food systems [2, 11, 12], and these interactions will differ across contexts and are localized in nature, providing an important entry point for informing adaptation options and responses [13]. Moreover, investigating in food security and food systems at a local level can aid in identifying (mal)adaptation trajectories that might endanger the long-term capacity of people to cope with and respond to climate change impacts[14, 15]

Indigenous peoples are particularly sensitive to climate impacts on food systems [16–18]. Emerging research has documented the experiences of Indigenous peoples and their food systems in the context of climate variability and extreme events, highlighting the importance of concurrent cultural, social, and economic conditions that can undermine or support food security [19–21]. Despite a growth in such research, however, the majority of studies have been conducted among Indigenous populations in high income countries [21, 22]. Research among Indigenous Amazonian peoples is nascent and has been identified as an important research gap- a research gap that will be particular important to fill in order to inform health-related climate change policies and climate-related health policy [21, 23]

Changing climatic conditions have already been reported in Amazonia, affecting the food security of local populations, however, consequences for Indigenous food systems are still largely unknown. Warming temperatures have been documented, with an average increase of 0.34C per decade recorded since 1980 [24, 25], along with more frequent and intense hydrological events [26–28]. In the Peruvian Amazon, increasing temperatures and more severe prolonged seasonal flooding and droughts are affecting agriculture and fisheries [29–31]. For Peruvian Indigenous Amazonian populations, however, initial research suggests that non-climatic drivers such as economic disadvantages and cultural marginalization are acting to magnify climatic impacts on food security [20, 32, 33]. Given that some Indigenous Amazonian populations have consistently worse nutritional outcomes compared to non-Indigenous populations in the region [34–36], climate change impacts on local food systems threaten to exacerbate health disparities. Yet this are of research is only nascent, and our understanding of how Indigenous Amazonian food systems interact with climatic and non-climatic changes to affect food security, nutrition, and ultimately human health is limited.

We contribute to filling this research gap by characterizing the complex food system, including the interacting climatic and non-climatic determinants of food security, and identify potential maladaptation trajectories within one Peruvian Indigenous Amazonian population. In doing so, we seek to promote consideration of long -term local food system adaptation of food systems in a changing climate.

## Materials and methods

### Conceptual and methodological approach

#### Food security and food systems

*Food security* represents a state of complete physical and economic access to sufficient, safe and nutritious food that meets a person´s dietary needs and food preferences for an active and healthy life [37, 38]. *Food systems* are understood as a set of human activities to produce, process, distribute, and consume food, and underpin the food security of human populations [39–41]. Given the high reliance of Shawi livelihoods on natural resources [42, 43] and recognizing the importance of food systems not only for nutrition but also for other health and socio-cultural components [39, 44, 45], this paper focuses on how disruptions in any of the four key food security components (i.e. availability, food access, utilization and stability) affect household and community food systems vulnerability to climate change [19, 20]. Under this framework, food *availability* is driven by food production, exchange, or distribution; a*ccess* represents the ability to consume preferred food sources; and *utilization* refers to the quality and the social value that people give to their food. At the same time, these food security components need to be *stable* over time to assure food security [40]. As such, food insecurity includes, for example, when food might be available but may not satisfy cultural preferences; food could be available and culturally acceptable, but not necessarily cover individual nutritional needs; or; food could cover nutritional needs and be culturally acceptable, yet availability throughout the year is not guaranteed.

#### Vulnerability approach

We structure our research using a ‘vulnerability approach’, which is common in the natural hazards and human dimensions of climate change scholarship [46–48]. This approach focuses on how various climatic and non-climatic factors interact to affect how people experience and respond to climatic risks and change, specifically drawing upon “contextual vulnerability” research that directs attention to the properties of the system and not climatic conditions *per se* [49]. Various disciplines have contributed to the evolution of vulnerability research, and this approach has been used by public health researchers to investigate climate-sensitive health outcomes, to prioritize vulnerable populations, and to evaluate adaptation options [50, 51].

Vulnerability can be defined as “the propensity or predisposition to be adversely affected” by the occurrence of climate change impacts[52], and comprises three main dimensions: exposure, sensitivity, and adaptive capacity [53, 54]. Exposure captures the nature of the climatic risk affecting the system of interest, in this case the Shawi food system. Sensitivity encompasses the characteristics of the food system that mediate climatic effects (currently or potentially), and reflect non-climatic drivers of food security. Non-climatic drivers are understood as key physical and social determinants such as socioeconomic conditions and existing health inequities [10, 55, 56]. Adaptive capacity represents the ability, resources, behaviours, or any other function of the food system that helps people respond to exposures and minimize impacts. In this sense, adaptive capacity is also an important *space* where non-climatic determinants interact with climatic risks face-to-face to offer opportunities for adaptation or which might result in maladaptive pathways [57, 58]. Maladaptive pathways refer to processes or conditions that exacerbate the sensitivity of human populations (e.g. social exclusion) or reduces options (e.g. erosion of knowledge about environmental risks) to respond to future climatic events. In other words, ‘maladaptive pathways’ represent changes or trajectories that are neither social nor environmental sustainable[59, 60]. The analysis of adaptive pathways, for example allows the identification of coping strategies to climatic or non-climatic stress that does not necessarily lead to sustainable long-term adaptations, which is crucial for populations that currently experience poor food security and social inequalities[59]. Adaptive capacity, exposure, and sensitivity are not mutually exclusive but are interconnected and interact across different spatial and temporal scales. The vulnerability approach recognizes the importance of sensitivity in driving the vulnerability of populations to climate change, thus highlighting the importance of non-climatic factors. For example, communities will be affected differently by identical flooding events (exposure) depending on the socio-economic conditions of each community and their support networks, the availability of backup support for resources, social conditions and a range of other non-climatic factors (sensitivity and adaptive capacity).

### Location and population

#### Demographic and climatic characteristics

**Fig 1:**
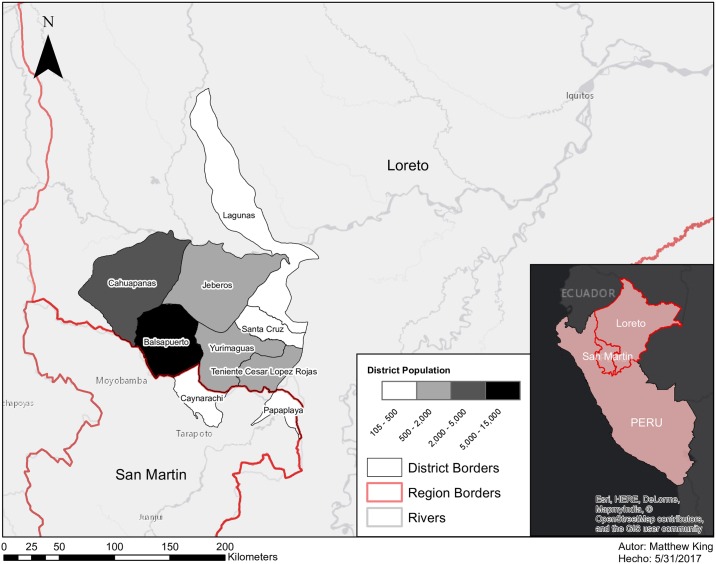
Peruvian districts and regions where Shawi people are located. Information about population obtained from INEI (2009). Locations where this study was conducted are not specified to keep the confidentiality of our participants. Reprinted under a CC BY license, with permission from Matthew King, original copyright 2017.

The Shawi are one of the five most numerous Indigenous peoples in the Peruvian Amazon [61], with a population of more than 20,000 inhabitants residing in more than 200 communities. Shawi settlements are located in the northern Peruvian Amazon along minor streams and rivers at the foot of the eastern slope of Peruvian Andes in Loreto and San Martin region (See Fig 1). Shawi communities have been exposed to multiple waves of visitors, missionaries and representatives of governmental initiatives, many of whom have focused on encouraging or compelling the Shawi to centralize their originally dispersed settlements and re-define their political and social organization, as well as their food production system [62, 63].

Evangelization, exploitation of natural resources, so-called “legalization” of native communities, and imposed education are among these changes [43, 63, 64]. Thirty years ago, for example, Shawi were described as an Indigenous society with two main production systems: one based on the utilization of the natural environment (e.g. shifting cultivation, hunting, and fishing) and a second related to trade and the market, but not necessarily through extended monetary transactions. Instead, some domestic animals or crops were bartered for goods or cash that were utilized for local livelihoods, such as fabrics to make blouses, batteries for radios, and ammunitions for hunting[63]. After centuries of interaction with the Peruvian-Spanish dominant society, Shawi continue to speak their own language and practice hunting, fishing, and subsistence farming, demonstrating cultural resiliency [64].

The Shawi territory has diverse altitudes and generally represents a sloping landscape. Most of the communities are located in regions of perennial non-seasonal flooding, although there are places that suffer periodic flooding in the rainy or wet season, especially those close to the edge of the larger rivers and down to the confluence with the Huallaga river [64]. Precipitation occurs throughout the year, although rainfall is particularly intense from December through April (wet season), with lighter rains from May through October (dry season) [65, 66]. High temperatures over the year vary with altitude from an average of approximately 19°C in the highest part close to Balsapuerto to an average of approximately 27°C in the lowlands close to Yurimaguas [66].

There are no downscaled future climate projections specifically for the region encompassing the Shawi settlements. Data from the nearest region, San Martin, however, indicate that annual average temperatures are projected to increase up to +1.8°C, with few changes to precipitations by 2030 (Ministerio del Ambiente, 2016). It is projected that rainfall will stay within its normal annual variability (+/-5°C), although seasonal precipitation changes are expected from -3% between December through March to +3% in April through June [67].

#### Shawi population

We worked with Shawi communities located along a river in the Balsapuerto district. To respect the confidentiality of respondents, and to prevent the possibility of reverse identification of individuals, we do not specify the name of the river or specific location of study communities. This paper reflects the results of qualitative research in two of the 18 communities along a river in this area (Fig 1).

Communities were selected after extensive consultation with Indigenous representatives at national and local levels, all of whom partnered with the researchers to develop an international research program, the Indigenous Health and Adaptation to Climate Change (IHACC) project. The two communities in the qualitative study were partner with based on their location between the most easily accessible (1hr road to the closest urban place) and the most distant community (2.0 hr road plus 6 hr on foot to the closest urban place) to reflect a diversity of access experiences in the region. Participants within communities were selected according to the location of their household to reflect a range of geographical access to the center of the community, where main educational and health services (if any) were located.

### Methodology

#### Ethics

This research was approved by the Research Ethics Boards at McGill University in Montreal, Canada and the University of Guelph, Canada, as well as the Institutional Ethics Committee at Universidad Peruana Cayetano Heredia in Lima, Peru, and followed principles identified in previous work in the region on the ethics of community engagement in research[68]. Informed consent was acquired from community authorities, and individuals, as well as from both parents in the case of adolescents.

#### Research approach

We employed a community-based participatory research (CBPR) approach to characterize the household and community Shawi food system and to investigate how climatic and non-climatic drivers affect vulnerability to climatic risk. Consistent with CBPR, all data collection methods were designed to allow for identification and exploration of emergent themes [69]. The use of multiple methods allowed flexibility in collecting key themes that may emerge through different data collection approaches, opportunities to triangulate results, and a chance to conduct iterative member checking with participants[69].

#### Data collection

We used semi-structured interviews, photovoice, transect walks, food calendar building, community engagement, and communal results dissemination meetings. Table 1 describes the aims of each method and a summary of participants.

**Table 1:** Description of methods, topics investigated and demographic characteristics of participants.

Method	Aim	Total activities	Total individuals
Transect walks with adults	Characterize crop ***production*** and ***processing***	2	2 (1 adult male; 1 adult female)
Food calendar building (focus group)	Characterize seasonal patterns of food ***production*** and ***processing***, as well as gender-age dimensions of these activities	1	8 (6 adult males; 2 adult females)
Participatory observation (home stay)	In-depth characterization of household experiences with food ***distribution*** and ***consumption***	1	13 (3 adult males, 2 adult females, 1 youth male, 1 youth female, 5 children, 1 adult female visitor). Community assembly suggested CZ live with one typical Shawi household for observations
Semi-structured (individual) interviews	Characterize food security, focusing on ***availability***, ***access***, ***utilization***, and ***coping mechanisms*** in the context of food scarcity over time	24	24 (9 adult males; 9 adult females; 4 elders; 2 teachers)
Photovoice (focus group)	Identify perceived factors that constrain or promote food security	3	17 (6 adult females; 4 adult males; 4 females and 3 male youth 13 to 16 y)
Meeting with community authorities and Shawi members	To disseminate preliminary results (e.g. food security status, food system, presence of external aid) and validate emergent themes (non-climatic drivers of food insecurity, access to local markets, educative desire)	2	First meeting: approximately 15 authorities from eleven communities. Second meeting: 4 authorities and 20 community members from two communities.

Data collection was conducted between June and September 2014 (Dry season in the Peruvian Amazon). The main investigator (CZ) and the Shawi research collaborator (GL) lived in one of the participant communities and visited regularly the second community to conduct the research.

All fieldwork was facilitated by one lead Shawi collaborator who was fluent in both Shawi and Spanish. The majority of participants preferred to converse in the Shawi language during research activities. Translations to Spanish were conducted in the presence of each participant to corroborate answers. Interviews were audio recorded, after participants provided consent. Reflective and descriptive journals were kept by the main investigator (CZ) [70]. Semi-structured interviews were conducted with key informants, including teachers and elders. Key informants were invited to participate, looking to reflect the diverse opinions of households located at different distances from the main community centre. Teachers provided complementary information about food sources received from governmental feeding programs at the school, and elders were included as the founders of those communities. Example questions included: What time of year is the best for eating well? What are factors that make this a good time for food? Do you remember a time when there was a severe food scarcity for you and your family? We did not explicitly ask questions about climate change, as we sought to understand more broadly how weather and seasonality affect food systems in the context of non-climatic conditions. Transect walks were conducted with two participants, who conducted tours of their lands. This approach sought to observe and discuss food production on the land and to explore constraints related to crop production and food processing. Two collective meetings with Shawi authorities and community members were organized to discuss preliminary findings and to validate emergent themes.

Detailed instrument guidelines are provided in the S1 File.

A photovoice exercise was conducted with three groups: females (23–37 y), males (28–37 y) and youth of both sexes (13–16 y). Photovoice is a participatory method that allows participants to answer a research question with pictures that represent issues or concerns relevant for individuals and the community [71]. It is often used with marginalized populations to provide insight into their internal conceptualization of their world and their problems[72, 73]. Two workshops were performed with each photovoice group. In the first workshop, the researcher explained the purpose of the exercise, the role of photovoice, the specific research questions, and how to use a digital camera. The research questions were “What helps you eat well?” and “What prevents you from getting food?” Participants were instructed to avoid taking photographs that included personal identifiers (e.g. faces) to ensure the privacy of community members. Each participant kept a camera for a period of 2 to 3 days, with photography aiming to capture visual pictures responding to the research question. During the second workshop, each participant was asked to select 5–6 photographs that they considered the most representative or important to answer the research question. These selections were printed and formed the basis of discussions in the second workshop.

Debriefing sessions were held every night with the principal investigator and the Shawi interpreter to identify emergent themes and to determine whether extra information or corroboration was needed over the following days [70]. For example, recognizing the need to capture food production cycles and key crops, we invited household members to collaboratively construct a calendar outlining crop types, seasons, and food production roles. Eight of the previously interviewed key informants were invited to build the seasonal food production calendar. Participants were asked to list main foods and then to discuss three questions: 1) what was the time period when those foods were available? 2) who was the individual mainly responsible for production or acquisition at household level?; and 3) what was the main purpose of producing or getting those foods?

Two meetings were organized with community members and authorities to disseminate initial results. During those meetings, we also validated emerging themes and verified community perspectives on possible pathways for adaptation (e.g. importance of education and constrains to market participation).

#### Data analysis

All data were transcribed and examined using thematic analysis [74]. Data were initially coded to characterize key elements of Shawi food security following the theoretical framework to explain food security under environmental changes. We then posteriorly characterized themes related to climate change vulnerability dimensions at the community level. *Exposure* characterized climatic risk to the food system (proxied here by weather and seasonality), *sensitivity* included social, environmental and economic drivers, and *adaptive capacity* considered individual, collective or external coping and response efforts. Initial themes from this framework were critically analyzed using an inductive pathway approach [58, 60] to identify key adaptive trajectories that potentially threaten short and long term adaptive capacity of Shawi households and communities. We then used narrative analysis for interpretation and presentation of the data [75, 76]. Narratives were contrasted with main themes identified in early stages to localize speakers’ information within the vulnerability framework. Narrative analysis allowed us to separate the researchers’ definition on the duality of *food security-food insecurity* from Shawi cultural definitions and perspective[77]. Narrative analysis also enhanced the process of language transcription from Shawi–Spanish-English by offering opportunities to re-evaluate and re-visit what participants *actually* intended to communicate[75]. We additionally drew on information from relevant literature about Shawi (primarily in Spanish) to validate, triangulate, and/or complement results.

## Results

### The Shawi food system

**Fig 2:**
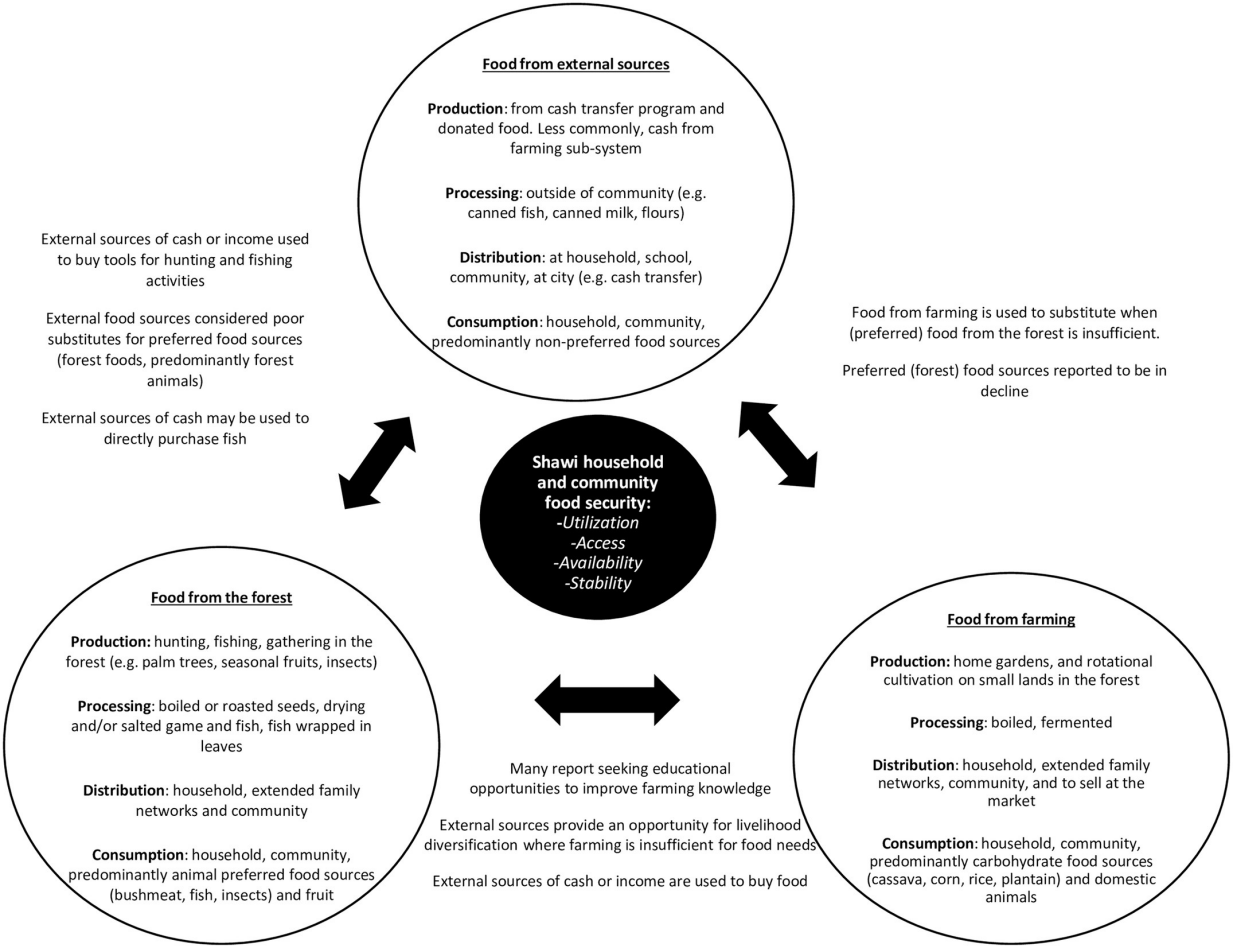
Food sub-systems among Shawi in two communities in Peruvian Amazon region.

Three main food sub-systems are currently ongoing and interact with each other in the Shawi communities participating in this study (Fig 2). These included: 1) wild food accessed from the forest; 2) food cultivated through farming; and 3) food acquired from external sources. Ongoing food acquisition from wild forest resources and food production in household gardens comprised more localized food systems, but also use of more distant land spaces in the forest that are adapted to produce crops annually and throughout the year. Cash conditional transfer programs and food aid distributed within the community or at school represented more recent and external components within the food system. Access to cash income to purchase food remained rare within the communities. Local Shawi food sub-systems were predominantly based on locally developed knowledge and technology, while the external food sub-system was generally sponsored by governmental initiatives, either by working within communities (e.g. school feeding programs) or in the closest city (e.g. to receive cash benefits).

#### Local Shawi food sub-systems

The forest was reported by participants as a space where hunting, fishing and gathering food were conducted throughout the year. Forest resources such as leaves and vines were additionally reported as important materials for food production, processing and transportation. For example, male youth described as a key theme “*plants that are needed to bring food to our home*,” where they explained the importance of some toxic vegetables to fish, leaves to wrap and prepare meat or fish, and vines to prepare baskets for transporting food. Participants documented techniques to identify animals for hunting, fishing and gathering in the forest (Table 2). Similar strategies and knowledge for accessing food from the forest have been reported among other Shawi communities and used by other Indigenous people in the Amazon [42, 78]

**Table 2:** Forest food system activities for Shawi.

Description of food activities performed in the forest	
Hunting	*Chapaña* is a hunting strategy involving hiding in the forest near wild fruits that are attractive to animals; this approach is used only during daylight and in quiet places
Fishing	*Huaca* (*Clibadium spp*. in Shawi A*kawa*) is a plant reported to be used to prepare a kind of paste for fishing, where the *huaca* preparation is placed inside a locally made basket and sunk into the water, usually in calm waters. The resulting toxic affect of *huaca* on fish is describe as temporary *Barbasco* (*Lonchocarpus spp*. *In* Shawi *Pena Nin*) is used for catching more diverse and larger fish species. When *barbasco* is used, typically multiple families within the same community participate and usually inform communities down the river to take advantage of the fish downstream.
Gathering	*Suri* (larvae of the order *Coleopterus spp*) and S*iquisapa* (ant order of *Cephalotes spp*) were also reported to be consumed and highly appreciated

Farming activities included the production of multiple crops and rearing domestic animals. Shifting cultivation was typically conducted on distant plots in the forest (30–60 minutes walking distance from the family home) called “*chacras*” in Spanish, while chicken and ducks were usually kept in backyards. Fruit plants including plantain, papaya, cocona and some palms tree were also kept in the *chacras*. This is consistent with previous studies, which have described Shawi local communal arrangements that distribute land to their founding family units for multipurpose use, including farming, hunting, fishing and house construction[43]. Cassava, plantain and sachapapa (*Dioscorea trifida*. a tuber in Shawi *Ma’ma yaraton or Ma’ma wiriton)* were reported to be the main crops harvested for self-consumption on farmed land.

#### External Shawi food sub-system

External sources of food comprised a variety of food types sourced outside of household or community production, typically received through external governmental aid or to a lesser extent through purchase. Some Shawi households received external aid in the form of cash income or food aid as part of public initiatives to increase food and social security of vulnerable populations in Peru [79]. A more detailed characterization of the three main governmental programs–the *Juntos* conditional cash transfer program, the *Vaso de Leche* food security program, and the *Qali Warma* school feeding program—and their contribution to Shawi food security has been reported previously [80]; one key finding included Peruvian governmental programs not aligning with Shawi food preferences, incorporating neither locally developed food sources or social institutions such as food sharing networks. Sharing networks were reported typically to be activated to protect vulnerable people such as elders or women pre- and post-childbirth, or during collective activities for producing food [80].

Some key informants also reported that they occasionally bought food by travelling to the closest city or from sellers that sporadically visit the community (*regatones*). We observed that *regatones* generally sold salty fish. One of our study communities had a small local store, and we observed eggs (not locally produced), canned tuna, cookies, rice, sugar, candies, beer and cigarettes were for sale. One key informant explained that neither cassava nor plantain was sold in the local store because “*everyone has it*”. No vegetables were available at the store, with the exception of onion and garlic.

#### Shawi diet

**Fig 3:**
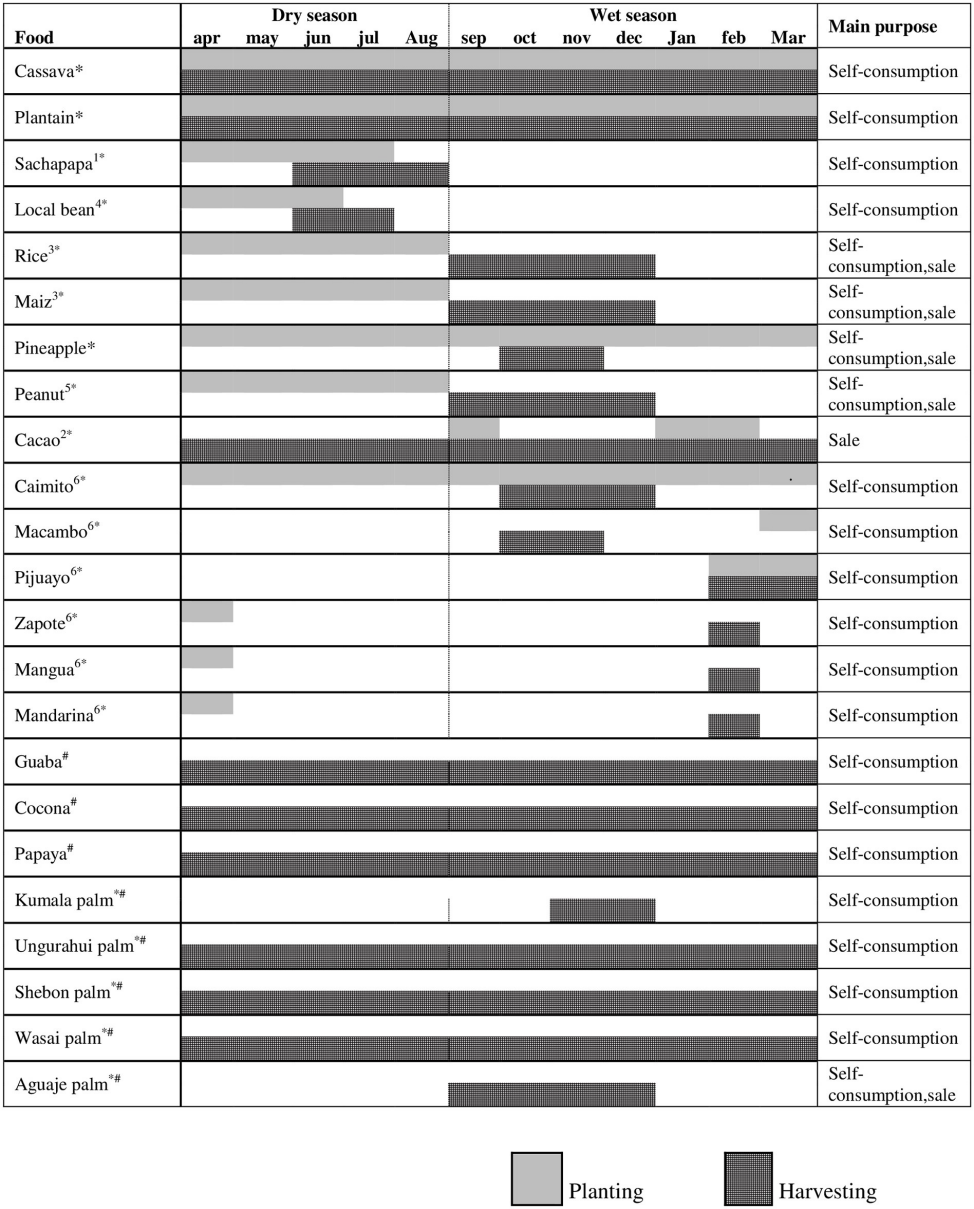
Availability of food over the year: Crops and fruits. 1: harvest a year after planting; 2: harvest three years after planting; 3: harvest 3–4 months after planting; 4: produces only once per year; 5: harvest four months after planting; 6: harvest five years after planting only one harvest per year. *Food from the farm sub-system; ^#^ Food from the forest sub-system.

**Fig 4:**
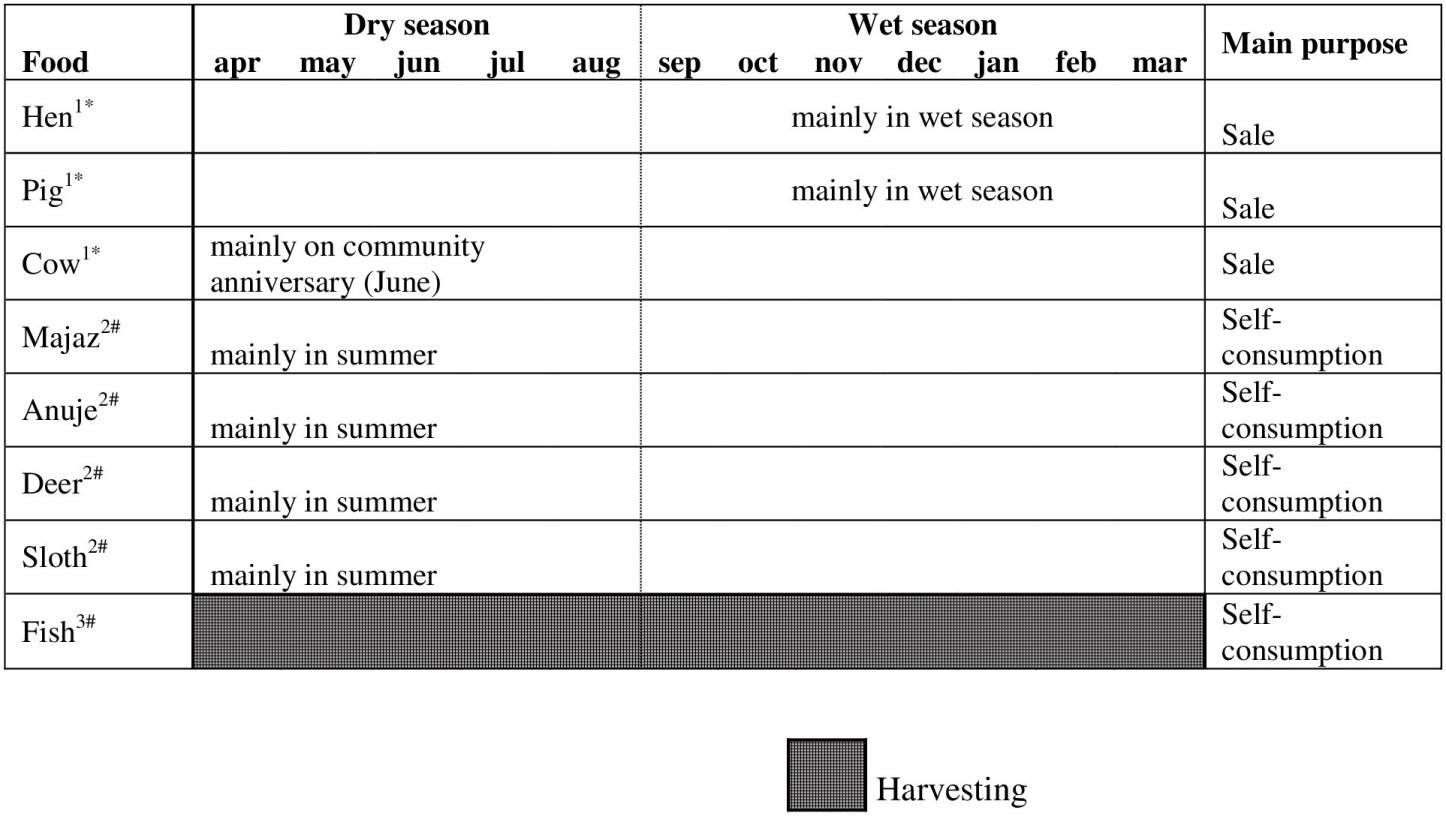
Availability of food over the year: Animals food sources. 1: Not eaten in the past; 2: Available in the past all year around, but specially in summer; 3: Only few species and small amounts. *Food from the farm sub-system; ^#^ Food from the forest sub-system.

According to informants, a typical Shawi diet would include a piece of cooked meat (bushmeat or fish) accompanied by boiled plantain and a locally made beverage, masato. Insects, fruits and other types of vegetables (e.g. local beans) were also consumed seasonally. Figs 3 and 4, illustrates the seasonal availability of different type of Shawi foods and their main purpose.

Annual food production comprises cropping, raising domestic animals, and accessing wild foods from the forest. Many crops followed a seasonal and/or inter-annual pattern of planting and harvesting. Crops such as maize and rice have shorter harvest cycles (3–4 months) and are harvested once a year (Fig 3). Cassava and plantain were considered to have moderate-length harvest cycles (up to 18 months); these crops were produced continuously over a year but require frequent weeding. Fruit trees were reported as long-term crops that take five years to fruit, and then fruit only once per year. Domestic animals were only consumed when there was “*nothing to eat*”–for example during winter when heavy rain constrains people’s ability to visit the forest or their crops to access food (Fig 4). Domestic animals were thus retained as an asset for consumption during difficult times, and not otherwise used for daily food consumption. Domestic animals were also reported as important for selling and access cash when traveling to the closest city to buy food, cover health expenses, education supplies and transportation fees.

Despite numerous recognized sources of local foods, participants reported that the Shawi diet was changing and it was reflected in the dynamic interactions among the three food sub-systems. First, there was consensus among participants that large and highly-valued forest animals, which represent the most preferred type of local food, were increasingly unavailable. In this context, the Shawi reported increasingly turning to minor, less preferred species such as rodents, small species of fish, insects and crustaceans (e.g. river small shrimps). Male photovoice participants identified “*food that is simple to get*” as a key theme, presenting a series of photographs depicting the type and amount of food that they were increasingly accessing to substitute diets when other larger and preferred species were unavailable. Photographs within this theme depicted rodents, fish, cassava and plantain, which were described as foods that were available but less desired. In contrast, photographs of larger animals such as deer, pecari, armadillo, monkey and alligator were seen as declining yet preferred. Other food sources reported as substituting for a decline in preferred forest animals included farmed foods or domestic animals, purchase, and external food aid.

Second, access to food aid with the external food sub-system was restricted by eligibility requirements, including childhood educational requirements (*Qali Warma*) and other household criteria for the *Juntos* cash transfer program. Donated food included sugar, rice, noodles, canned tuna salt and oil. Cash obtained by selling products from farming was reported to be used to buy food, although participants generally indicated that other expenses—particularly school supplies—were often prioritized over food. Third, key informants reported using cash to buy tools to support hunting and fishing to access preferable food sources or to directly acquire fish or game meat. This pattern of using money to favour, directly or indirectly, preferred food sources was consistent with researchers working elsewhere with other Shawi communities along different time periods[43, 63].

#### Shawi perceptions of food security

**Fig 5:**
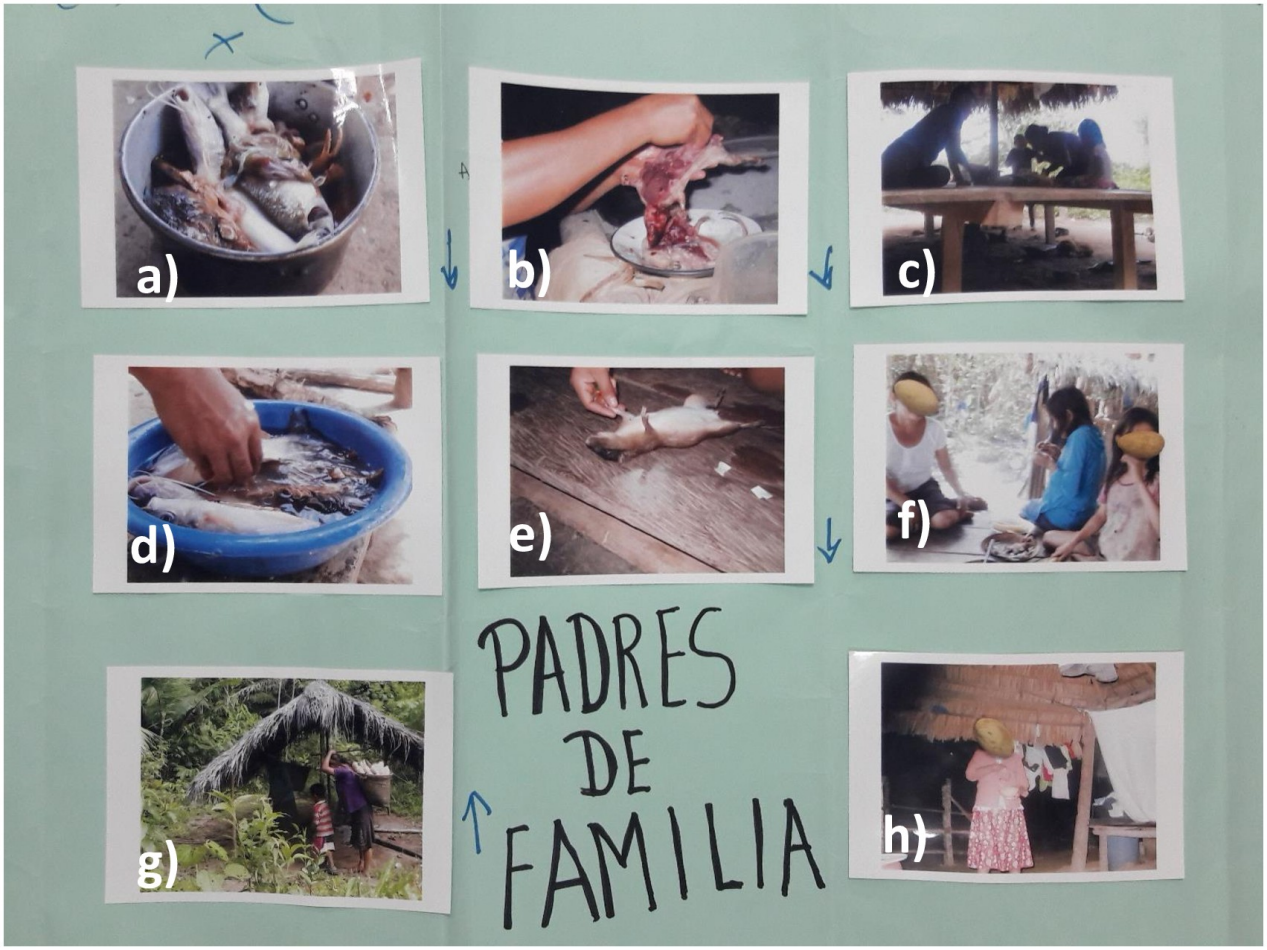
Poster created by Shawi male participants in the photovoice workshop to discuss current food security. a) fish brought from the local river in the forest is ready to cook b) a women cleaning a small rodent hunted by one participant, c) household members who will share the food d) cleaning the fish with water from the river e) a small wild rodent called “sacha cuy” in Spanish, f) household women sharing one plate of food, g) a women harvesting cassava on the family farm, h) a grandmother who was unable to produce much food, but was able to prepare and shared her masato, a Shawi Indigenous beverage, made of cassava with the rest of her family.

There was a strong culture of collectivism, gender participation and local food preferences associated with Shawi food systems and food security. For Shawi participants, food security represented a state where not only individual food needs were satisfied, but also when households and community members are able to bring, prepare and share enough, preferable and safe food for a happy life. Fig 5 shows a poster developed by Shawi adult male participants in the photovoice workshops. They used this poster to discuss different components of their current food security. The poster included photos of food (e.g. fish and small rodents) but also pictures of family members sharing food, including an elder female preparing masato as an invitation to the rest of her family. In addition, both groups of adult women and men in the photovoice activity concurred that because wild animal food sources are increasingly scarce these days, they felt sad and concerned that they will not be able to provide food to their families in the future. The adult female participants in the photovoice explained:

“*When we eat [local foods]*, *we are happy*, *however these days we feel sad and think that fish will disappear*. *We think that in the future we will have everything from our gardens*, *but not fish*”.

(Adult women participating in photovoice)

Participation in food system activities was a key component of Shawi food security. Food system roles and responsibilities within a household were reported as clearly patterned by gender and age, and often involved collective activities (Table 3). Photographs identified and discussed during the photovoice exercise indicated that adult men predominantly engaged in hunting and fishing for animal food sources while women mainly participated in gardening/ cropping activities focused on subsistence cassava cropping and food preparation at home. Collective activities to produce, distribute, and consume food were described as an important component of the local Shawi food system. A summary of the photovoice results is included in S2 File.

**Table 3:** Food system activities patterned by age, gender, and collective participation for Shawi.

Food activity	Female	Male	Youth/children	Collective participation
Fishing	Catching minor fish species using toxic plants and with bare hand technics. Processing and preparation of fishing food such as *Shikana (wrapped* dry-out fish)	Use of fishing tools and toxic plants	Children know how to fish with a hook. Youth help to prepare plants for fishing	Practice with other relatives or close friends. Also, an activity where the whole community members can participate
Farming	Production, processing, preparation and serving, specially cassava based food e.g. *masato*. Rearing chicken and ducks	Cash crop production: maize, rice and sometimes plantain. Planting and harvesting larger fruit trees	Harvesting beans with their mothers. Adolescent girls taught food knowledge by their mother	Among relatives and close friends within the community (e.g. *mairesu* in Shawi).
Hunting	Normally do not participate	Use of shot guns and techniques for hiding/tracking in the forest	Adolescent males taught hunting knowledge accompanying their father	Among relatives (grandfather, son in law and grandson) and close friends within the community.
Gathering	Family activity	Family activity	Collecting insects from the forest	Among relatives and close friends within the community

The two transect walks were focused on communal activities oriented towards preparing the land for the cultivation of maize (with a male participant) and harvesting of cassava (with a female participant). Communal activities were reported by key informants as critical to efficiently and effectively organize the necessary labour for time-sensitive initiatives.

#### Local experiences of climate variability and change

For Shawi participants, annual climate variability reflected in seasonal precipitation was critical to developing local food sub-system activities on the farm and in the forest. Even though they did not report on direct effects on the external food sub-system, precipitation was highlighted as having implications for attending school, which was a pre-condition to receive governmental aid. As climatic variability and change reflect long-term averages of meteorological variability, Shawi observations reflected weather and environmental changes that occurred locally and on shorter time scales, and that were, to varying degrees, affected by longer-term climate change.

Annual variability of precipitation was important for crop food production and affected forest food accessibility. The dry/summer season lasted from about May to September, while the wet/winter season lasted from October to April. Key informants explained that the dry (summer) season—when rainfall decreased—was a more productive time for crop cultivation. During the dry season, community members typically engaged in cleaning, burning and planting crops. Key informants explained that some rain was necessary to soften the soil to plan cocoa trees and palms species so that they will produce fruit in the winter months. Rice, maize, plantain and several fruit trees, however, required extended periods free of rain to allow for clear cutting and burning of vegetation for effective sowing of crops. In addition, during the dry season when water recedes and water bodies were shallower, it was reported that fish are easier to harvest. Walking on forest paths to travel distances and hunt wild animals was also easier during the dry season. In contrast, during the heavy rainy season (winter), crop production was poor, access to game and fish sources from the forest was difficult and fulfilling food aid eligibility requirements was often challenging (e.g. school attendance during heavy rains, transportation on muddy paths and crossed streams).

Shawi participants have noticed warmer temperatures and changes in precipitation and reported concern regarding the potential effects on their farming sub-food system. Adult women in the photovoice sessions explained that when the rainy season comes early, they were unable to effectively clear and prepare the land for planting their main staple crops, cassava and plantain. Women used fire to burn their fields in preparation for planting; early or extended rains during the dry season interfered with this local crop preparation activity. Our results are consistent with reports from Shawi communities elsewhere, with observations of changing and increasingly unpredictable weather affecting land preparation, burning, and crop production[42].

The Shawi reported knowledge regarding water river variability and what do to in response to flash flooding events that regularly affected their communities. Key informants reported that the local river experienced sudden level changes, with flash flooding typically occurring at some point in the beginning and at the end of the wet season, between September-October and March-April respectively. They described water increases that rapidly lead to water overflow, with flooding typically lasting a few minutes to a maximum of one hour, and reaching only few centimeters high, thus affecting low lands and some parts of the center of the community. Elder participants mentioned that they did not deforest, crop, or build their homes on these low lands or close to the edges of the river to prevent risk of flooding.

Elders clarified that flooding was “*serious*” when the water covers fields (e.g. plantain, cassava, peanut, or maize); this level of flooding had the potential to damage crops and/or housing, as well as injure or kill domestic animals. The elders recalled the flood of 1995 as one of the most severe flood events in the last decade. In addition, key informants also reported that living and cropping in higher lands was seen as a better place for secure cultivation and safety during flood events. One female elder recalled what she and her family did during the 1995 flood:

The water was up to our knees. Because it was during the daylight, we could run to the highest part of the community, carrying our possessions such as cloths and domestic animals. We did not lose anything important, only used mocahuas and callanas [clay locally made vessels], and our house was moved by the water flow only a bit

(AA013, female).

Another key informant reported that her family did not lose anything important during the 1995 flood. The water reached her house but did not affect her crops because her family always farmed on high ground. One younger participant declared that the 1995 flood reached his house while his parents were in the forest. He had to travel by canoe to cross the river and to reach the highest part of the community for safety.

#### Non-climatic drivers of Shawi food security vulnerability

Participants reported facing several years of food insecurity characterized by a decreasing availability of wild animal food sources. Food insecurity was attributed to a combination of socio- and environmental factors affecting local food systems, namely: a rapidly expanding population, changing settlement patterns, and natural resource degradation affecting river shorelines and wildlife.

Younger key informants noted that they did not remember a time when game and fish were abundant. Older adults and elders consistently reported a marked decrease in game and fish, noting that these changes have occurred over the past 20–30 years. For example, one adult woman reflected:

When I was a child, I remember my parents had enough game. I saw it. Even when I was an adolescent I had food, but when I got married and my oldest child was born [24 years ago], I noticed the difference. Sometimes you find food [game], others times you don’t

(AA016 female, 44 years old).

Key informants and photovoice participants consistently reported that the population and number of new Shawi communities is increasing, with implications for availability and access to wild animal food sources:

When I was a child, there was animal food every day. Although, we had some [meat] at least until I was fifteen years old. Then more people started to appear and they eat the animals

(AA014, male, 34 years old).

Elders, in particular, agreed that an increasing local population was associated with decreasing animal food sources. They also attributed animal food availability in the forest to a shifting settlement pattern—from more dispersed to more centralized—with higher densities of people competing for access to animal sources of food. A perception of disrespect of Shawi norms was reported by Elders when discussing the expanding population. One Elder key informant explained:

Only my family used to live in this zone, we had to walk far to find another house. This is the reason that we used to have a lot of bushmeat. Before, nobody crossed your path. If a person crossed your path, it means that you did not deserve respect and were a source of conflict. It was like a rule. These days, [rules] have disappeared; there are no rules. Everyone walks the same path that you are walking

(AA011, male 70 years old).

Furthermore, more people were carving out new plots, in many cases via deforestation along the river shore. Deforestation and erosion of river edges has been associated with changing river structure (wider and shallower), and have been leading to new habitats that are not suitable for larger fish. In response to difficulty catching fish, participants reported that there was increased use of poisoned fishing techniques that involve *barbasco* (e.g. rotenone poison based) which were exacerbating damage to the river ecosystem. As presented in Table 2, *barbasco* utilization was typically intended to promote collective fishing activities and at certain time of the year when fish were more abundant. Key informants reported concerns that overutilization of poisonous plants might further diminish the availability of remaining wild river resources.

Local authorities concurred with these trends during results dissemination meetings, noting that food security in communities was affected by an increasing population reflected in the creation of new villages. In addition, authorities explained that accessing cash income to pay for school was a key driver of deforestation, with implications for natural resource degradation. Despite this, education was widely perceived as an important option for livelihoods diversification and food security in the future.

Shawi youth participants in the photovoice exercise were also aware of the very low food security that their households were experiencing. They referred to the impact that more people might have on the availability and access of wild food sources in the future. Shawi youth expressed a desire to have fewer children than their parents (1–2 vs 4–5 children). They specifically asserted that they wanted to “*feed their children better*”. During the photovoice workshop one youth expressed:

*In the future*, *there will not be bushmeat*, *we probably will handle more money*. *We are going to be fine*. *We are going to have fewer children*, *in that way we will be able to feed them better [a piece of game or fish for each kid]*. *We will keep our crops like cassava and plantain*, *of course if we plant and take care of them*.

(Youth participant in a photovoice workshop)

### Interactions of climatic drivers with non-climatic drivers

Shawi households and communities were exposed to multiple drivers affecting their food systems and shaping their current and future vulnerability to climate change. Climatic and non-climatic drivers interacted at two levels to shape Shawi food security vulnerability: one *proximal* level that immediately threatens the local capacity of Shawi households and communities, and a second *distal* level that will compromised further local responses to climate change by reinforcing proximal drivers, and thus potentially increasing the vulnerability of Shawi to climate change risks.

**Fig 6:**
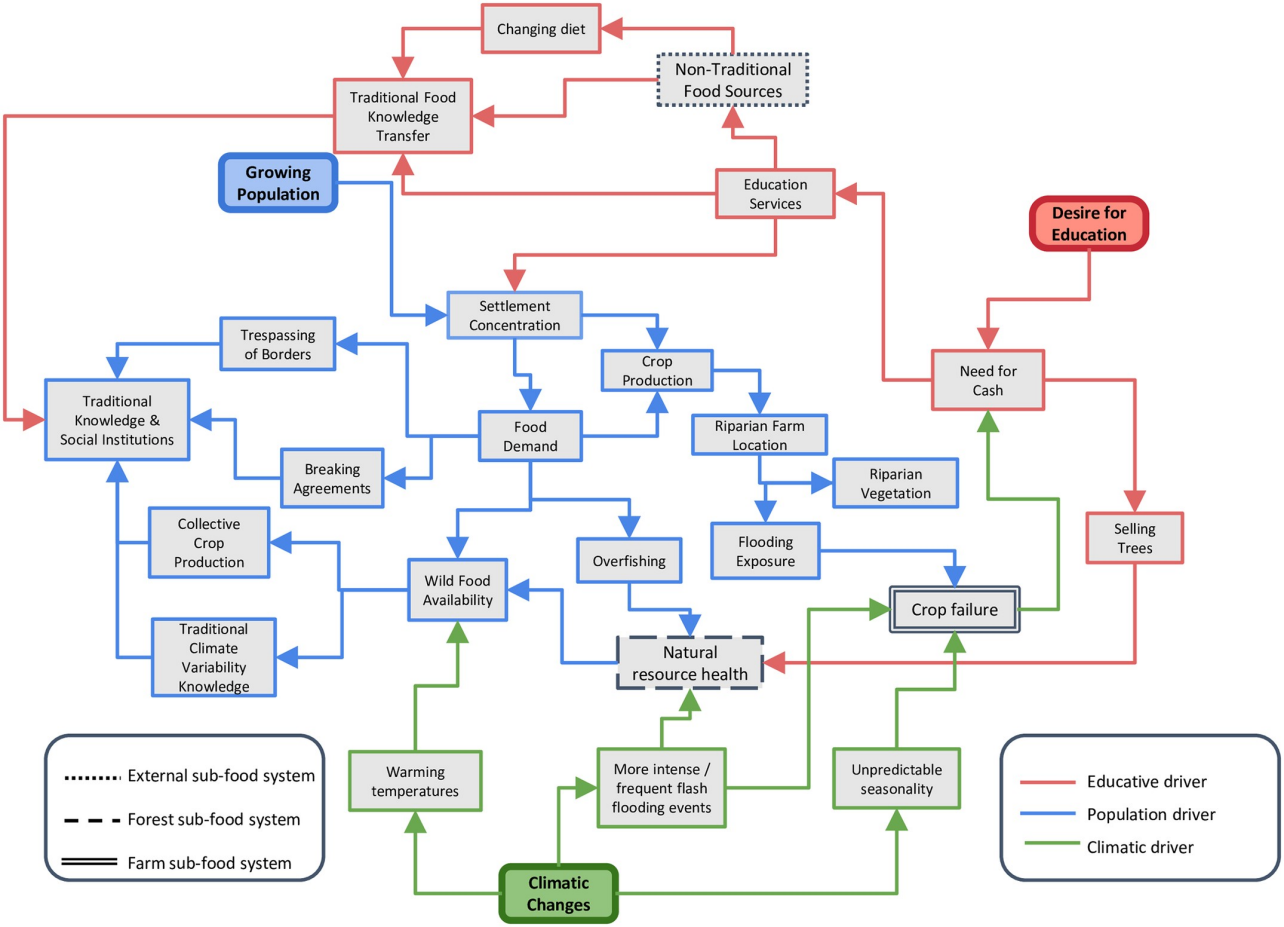
Proximal drivers of Shawi food security vulnerability and their interacting effects in each of the Shawi food sub-systems.

**Fig 7:**
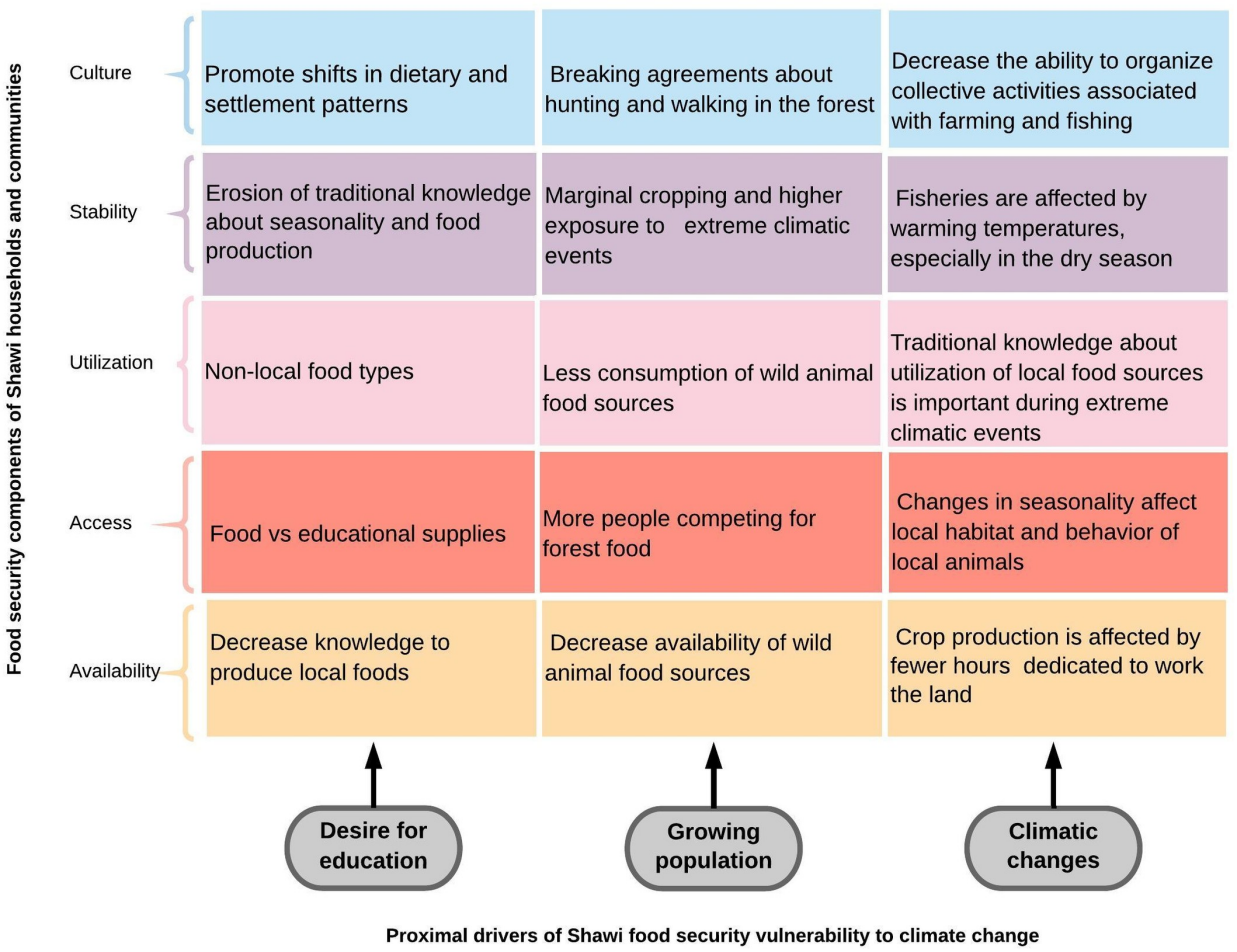
Effects of proximal climate change vulnerability drivers on each component of Shawi food security.

Fig 6 illustrates the interactions between the three-identified key proximal drivers of Shawi food security vulnerability and their impacts on the Shawi food sub-food systems while Fig 7 provides examples of the effects of proximal drivers on each component of the Shawi households and communities food security.

#### Proximal drivers of food security vulnerability to climate change

Demographic drivers directly affected food availability and exacerbated the Shawi food system sensitivity to climatic risks. Demographic changes in the context of concentrated community settlements were elevating pressure on wild food sources and extending the spatial extent and patterning of farming by using river edges for crop production, which in turn increased natural resource degradation. Cropping near the river was recognized as exacerbating the exposure of people and crops to climatic hazards Despite the risk that river edges represent for crops, key informants described the need to plant new plots close to the edge of the rivers because of an increasing dependence on farmed food. This marginal cropping was reported to occur in the context of limited opportunities to access cash and decreasing wild animal food sources. In parallel, lower tree density arising from deforestation for cropping along river edges negatively affected water dynamics and compromises fishing availability.

As long as fishing and other wild food sources were less available, the local Indigenous mechanisms for organizing collective work for crop production and collective fishing were compromised. The increased number and density of food insecure people seeking food in the forest were also encroaching household land boundaries and affecting Shawi social norms. This trajectory leads to diminishing local social institutions and the knowledge associated with the production and use of wild food, which is essential to responding to climatic hazards events. Furthermore, people were building new crop land on river edges, which increased their exposure to crop failure due to more frequent flooding events (Sistema Nacional de Defensa Civil-Balsapuerto, 2014, Sistema Nacional de Defensa Civil-Balsapuerto, 2015, Sistema Nacional de Defensa Civil-Balsapuerto, 2016). Consumption of wild food combined with increasing population was compromising the availability of wild food, implying that this cycle was unlikely to be sustainable.

The second proximal pathway was driven by the desire of formal education (Fig 5). Desire for educational opportunities presented a trade-off between investing in education (purchase of school supplies and transport), maintaining food security, and facilitating illegal logging. Community authorities recognized that even when local regulations exist to prevent indiscriminate deforestation, poor access to cash income for children’s school expenses (e.g. parents’ association fees, school supplies, transport) led them to agree to sell trees from their land to visitors, who were frequently illegal loggers. Selling to illegal loggers implies that benefits were not guaranteed and there was greater chance of exploitation. Increased education, in its current form, may conflict with and compromise Indigenous Shawi knowledge, as children spend less time with their parents, and youth leave the community for school, often losing knowledge of the land, food production, and local food consumption. Desire for education was also driving changes in settlement patterns with impacts on people’s access to, and relationship with, wild food sources. Other researchers have highlighted access to schools and education as a key driver of changing settlement pattern among Shawi people [43, 63]

Third there are changes in the local climate system, including warming temperatures, changes in seasonality, extreme precipitation and more frequent flash flooding events that interacted with the other two non-climatic drivers. Recent perceived alterations in climate variability were affecting the Shawi food system and compromising availability of food. According to our participants, warmer temperatures affected soil and local water bodies, and were particularly problematic during the dry season when most people needed to work outside to prepare the land, whereas river and creeks were the closest sources of animal foods. During transect walk activities, participants noted that temperatures have warmed in recent years, making it difficult to spend long working hours on the land. Previous researchers working with Shawi participants have also reported that warming temperatures were affecting water quality, which in turn affected the availability of fisheries in the dry season when water river decreased; temperatures were hotter and the expected minimum precipitation was perceived to decrease since 2005 [32]. In addition to changes in temperature and precipitation, respondents reported that the predictability of seasons was also changing, with winter perceived as starting earlier than usual in the year. Local “*August winds”* that marked the beginning of the winter season were reported as occurring one month earlier than normally expected. According with local observations, not only was annual variability is changing, but also the behaviours of the fauna, which implies more uncertainty around the availability and stability of food. For example, *zikizapa*, an edible specie of ant, appeared early in September on 2014, compared to its normal expected emergence in October or November.

One key informant stated:

*“In the past*, *we had a specific time when the summer began*, *when the winter is*. *Today there are days when it rains during the summer*, *it rains for a whole week*! *It is no longer in order*, *it is in disorder*”

(B001, male 25 years old).

For the Shawi, social activities and celebrations were based on wild food (to compensate for the collective labour carried out in the community). Preparing the land for cultivation by practicing collective activities—*Mairesu*, for example—was considered critical to avoid rains during planting. Early arrival of rains and unpredictable seasons made this collective capacity even more crucial. Since wild animals were central to preparing meals to organize this activity, the scarcity of game and fish compromises the ability of families to organize social activities around land cultivation and food availability from the farming sub-system is thus also affected. Shawi participants from other communities have also reported on the importance of game and wild fish for their community socialization[43]. Moreover, food sharing networks are embedded predominantly in the wild food sub-system and to a lesser extent the farming sub-food system, but not at all within the external sub-system.

Indigenous knowledge about food species and climatic characteristics were reported critical to cope with the impacts of climatic stresses. Wild food was a critical back-up when natural hazards destroyed crops or other food: the wild food system was thus an important safety net. Key informants explained that in 1995 because of intense rain in the highlands at the head of the river, a slope of the mountain collapsed, causing a mudslide. This mudslide was also reported by Peruvian authorities in the Balsapuerto district [81]. Participants explained that after this event they turned to the forest and wild food sources as a substitute for the damaged crops. Recognition of multiple plant varieties, animals, and wild fungus was critical to accessing food as part of the communities’ coping strategy to access food during the 1995 flood and mudslide. Key informants mentioned that they were able to survive on typical forest foods during this time, including: macambo (*Theobroma bicolor)*, cocona (*Solanum sessiliflorum)*, churo (*Pomacea maculata*), congompe (kind of snail), callampa (wild edible fungus), suri (multiple species of larvae) and chonta (multiple species of heart palm tree).

#### Distal drivers of food security vulnerability to climate change

**Fig 8:**
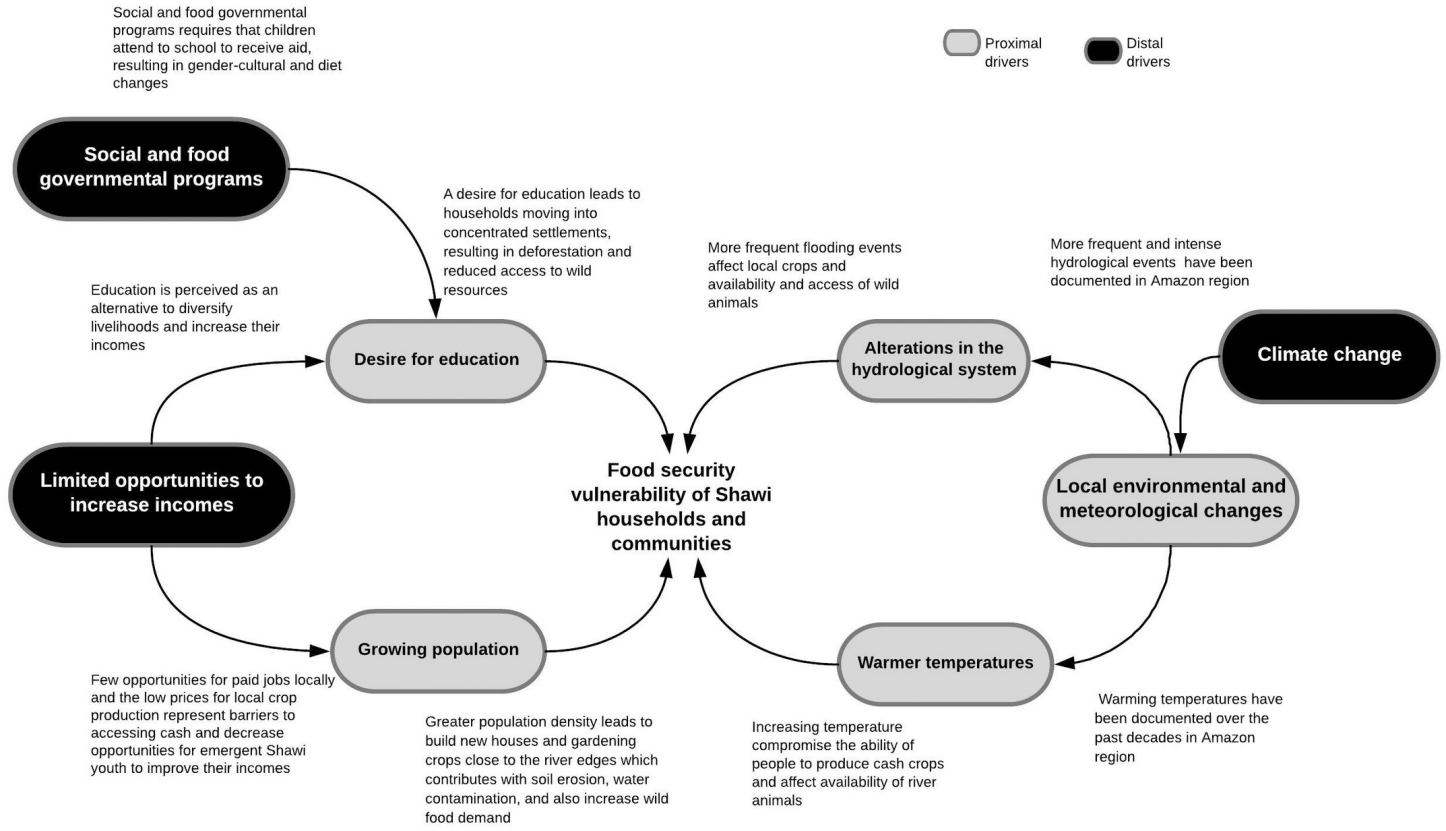
Distal drivers reinforce proximal drivers to shape Shawi food security vulnerability to climate change.

Key distal drivers were associated with the regional and national Peruvian economic context and included limited opportunities to increase local incomes and social and food governmental programs currently on going in the Shawi territory (Fig 8).

Participants identified cash crops as one important alternative to generate cash income, although many Shawi reported concern that their crops garnered very low prices at the closest market where prices were generally imposed by buyers. Shawi small farmers visit the closet city to sell their food products to wholesalers. Wholesalers were intermediaries who re-sell Shawi foods to higher price to local stores and urban customers. One key informant highlighted the case of the *sacha inchi* (*Plukenetia volubilis*), for which prices had fallen dramatically; 10 year ago, this product sold for 10 soles per kilogram and, at the time of the data collection, garnered only 1 sol per kilogram. As a response to low cash income from crop sales, and based on their past experiences, Shawi key informants reported that most of their food production is now for consumption in the home. Elders reported that during the 1980s, they were involved in rice production in their own lands, although low prices in the closest markets currently discouraged them from pursuing rice production. Larger crops of maize were a notable exception to this, as were times when fruits prices increased sufficiently in the closest city to justify the sale of some surplus. Access to cash from the farming system was thus highly unstable over time. One male interviewee explained his decision to sell plantain:

“*All year-around we have plantain*, *however there is a time when the price is high*. *When a bunch of plantain has a good price*, *we sell it at 15 soles*. *Other times the price is low*, *around 7 soles*”

(AA021, male 50 years old).

Economic drivers intersected with education and demographic pathways (Fig 8). Few opportunities for paid jobs for educated returnees and the low prices for local crop production also represented barriers to accessing cash and decrease opportunities for improved food security. Continued education was seen as increasing access to paid jobs, and was perceived as one alternative to improving incomes. However, community authorities highlighted that even when they supported their children to attend school, usually travelling to distant communities to access secondary school or better training, few job opportunities were available in their territory for returning students. Community leaders expressed frustration that there are few employment alternatives or local jobs:

*To support agriculture that is an alternative to Shawi youth*, *that is the only alternative*, *where young adolescents are paralyzed with completed secondary studies*. *There is no alternative for them*. *We know when youth achieve a high level of education*, *they share [the benefits] with their family*. *If s/he has something [an income]*, *s/he will share with his/her family*.

(RD, 001, June 2014)

Desire for further education was also expressed by youth participants during the photovoice activity. Youth participants discussed the importance of achieving higher education as one of their main goals and options to learn new techniques to improve their farming production in the future.

A second distal driver was the presence of external aid from Peruvian governmental authorities (Fig 8). External aid in the form of school feeding programs or cash conditional transfers in theory can strengthen the educational pathway. Access to educational opportunities is a corollary of the conditional *Juntos* cash transfer program[79]. *Juntos* provides economic incentives ($ 60.00 US bi monthly) to poor and extremely poor households under the condition that household participants must complete various requirements including 85% attendance at school for children 6-14yrs[79]. At the same time, the *Qali Warma* feeding program delivers food only to children who attend to school. This implies that external aid could exacerbate a maladaptive trajectory by promoting education in a place where economic job opportunities are limited for educated Shawi and where access to cash to cover expenses for higher education is also scare. External food and aid operates under a Peruvian economic model that does not support local capacity, potentially increasing vulnerability because it erodes social cohesion by ignoring Shawi culture such as language, food preferences, and local Indigenous social institutions (e.g. sharing food and gender roles). Food sharing networks for example, are widespread among Shawi communities, and ensure food for vulnerable sub-populations (e.g. Elders) in difficult times. Food sharing networks were mainly based on sharing of local food sources [80]. However, food aid used non-Shawi or non-preferred foods, and targets individuals within households with negligible consideration of cultural norms around sharing and food production. Shawi farming and forest sub-food system activities were based on gender participation, for example, where males were associated with hunting and women with gardening. Given that bushmeat was decreasing, males were also losing their main social activity *vis a vis* food provision, implying that males were facing the Shawi cultural equivalent of unemployment. However, national food and cash governmental programs target mainly women and thus have limited consideration of the eroding social hunting norms among Shawi men while simultaneously increasing labour and responsibility for women.

## Discussion

This paper aimed to characterize the food system of the Shawi of the Peruvian Amazon, climatic and non-climatic drivers of their food security vulnerability to climate change, and identify potential maladaptative pathways. Our investigation of the Shawi food system highlights how the natural environment, culture and socialization are fundamental components of the Shawi food system. Wildlife, local crops, collective activities, gender roles, and emotional connections comprise Shawi household and community food security; and represents important elements to understand complex interconnections between climatic and non-climatic stressors. Indeed, our vulnerability analysis indicated that multiple drivers were interacting to determine current Shawi food insecurity. Some drivers result from socioeconomic events in the past century, while many others were ongoing. The investigation of proximal and distal drivers of vulnerability has allowed us to identify potential maladaptive pathways that, unaddressed, could increase vulnerability to climatic and other stressors.

To be sustainable and equitable, it is important that climate change adaptation options are rooted in particular socio-cultural contexts [60, 82]. Sustainable adaptations are particular important for historically social excluded populations who depends on highly susceptible ecosystems and populations [83] such as the Shawi and Amazonia. We present two key findings with implications for designing sustainable adaptation policies.

### Maladaptative pathways related to social and food interventions

Maladaptive pathways emerged when Shawi social responses interacted with Peruvian socioeconomic policies. For example, Peruvian social and food policies could have unintentional consequences for Shawi food security and could potentially will increase the susceptibility of Shawi to climate change through the erosion of Shawi social cohesion and collective institutions. Similar work in Brazil, for instance, illustrated that even tough anti-poverty social programs that increased cash incomes did not lead to a significant increase of the adaptive capacity to respond to climatic risk, mainly because those programs did not address the multiple non-climatic drivers of long term social vulnerability [84, 85]. Additionally, Peruvian policies were reinforcing a proximal educational driver was is associated with the expectation of increasing incomes and diversifying livelihoods. Educational desire has emerged as a key proximal driver of vulnerability of Shawi food security. Education did not necessarily lead to job opportunities and yet is having a substantial impact on food insecurity. The sacrifices made for educational opportunities—prioritizing expenditure on education over food, facilitating deforestation to access cash, adjusting settlement patterns to be closer to schools, reducing access to wild foods, supplementing with non-Shawi food sources, and resulting in a loss of Indigenous knowledge among youth—have to-date shown little benefit in the form of increased job opportunities or improved food security for Shawi.

The role of education as a mechanism for enhancing climate change adaptation, widely promoted in the general scholarship [86, 87], requires further investigation in the context of Indigenous Amazonian peoples. Access to formal education could serve to build social capacity (e.g. better health and poverty reduction) to respond to and recover from to any future climatic risk. Education can also increase the awareness about risk perceptions and can improve the ability to take actions when facing a disaster [87]. Working with a population of low income settlements in El Salvador and Brazil, researchers found that the level of formal education affected the likelihood of living in a risky area and that those with more education identified greater options for recovery after a hazardous climatic event [88]. This same investigation also found that education worked as a mechanism for recovery only where formal employment was available [88]. This suggests that improving access to education in and of itself will not guarantee that communities will be more prepared to respond to climatic risks. For example, one study identified literacy as a key ability to access information and aid from governmental initiatives [89]. For the Shawi, this would translate into the causal expectation that education would improve their Spanish and thus facilitate better access to governmental aid. However, this assumes that the benefits of education will outweigh any potential losses in local and Indigenous knowledge about preparedness and response to risk. Educational opportunities are likely critical for future generations of Shawi but should be developed in a way that does not erode existing coping mechanisms for food security and does not compromise food sources, for example culturally and nutritionally important food types [44, 90–93].

### Past and present experiences are shaping the future adaptive capacity of Shawi household and communities

Past-present trajectories of vulnerability associated with demographic changes and environmental degradation have being reported as important for future adaptation among Pacific Island populations where the “desire to make money” has resulted in the development of more cash cropping which in turn has eroded the social cohesion and reduced subsistence resources [57, 58]. Similarly, a demographic driver related with a less mobile settlement pattern and increasing farming practices was identified as a key proximal pathway for Shawi households and community food security. The Shawi have consistently increased their population over the last half century [63]. Improved access to health services, particularly vaccination, and the erosion of Indigenous fertility regulations (e.g. polygyny, marriage rules, contraception), have been hypothesised as key factors in the recovery of Indigenous Amazonian populations after major population declines following encounters with Western societies [94, 95]. Although we did not find updated Shawi data on infant mortality, the last Peruvian census (2007) indicates that the Shawi population was relatively young, with higher fertility rates compared to the Peruvian average (7.7 vs 2.6 children per women) [96]. This implies that the Shawi population may continue to increase in the future. Youth participants indicated that they intended to have fewer children and this intention may represent the potential for declining fertility rates if barriers to accessing appropriate and effective contraception planning are addressed.

Similarly, past and current Peruvian policies were associated with reported changes in community settlement pattern and interact with current Peruvian economic development policies (e.g. infrastructure and agroindustry). For example, a Peruvian law (D.L. 20653, 1975) was dictated to legalize native communities and to provide incentives (e.g. economic aid and loans) to promote agricultural practices [97, 98]. The law was intended to provide land ownership and promote economic participation among Indigenous populations. Despite this, the law has resulted in the fragmentation and reduction of ancestral Indigenous territory, consequently limiting Indigenous public access to land and to wild food system activities [42, 99, 100]. More recently, surrounding areas of Shawi settlements, in the San Martin region for example, were impacted by the promotion of bio-combustibles production [98] and the development of a road for transportation [101], with the Shawi expressing their concern about future land tenure conflict, immigration, deforestation, and reduced game availability [43].

So long as Shawi communities are losing their ability to migrate within their territory, dispersion as a coping mechanism to respond to extreme climatic events will be also threatened. Dispersion into the forest to access wild foods under extreme climatic events has been reported among the Yanomani people. The Yanomani turned to the forest to access food after an intense fire destroyed their crops during an extreme drought reported in the Amazon (1972–1973) [102, 103]. At that time, availability of forest wild food among the Yanomani was described as a reliable and important resource to supporting a *“nomadic existence*” [102]. Our results concur with reports from elsewhere in Latin America, suggesting that alteration of settlement patterns associated with increasing dependence on agricultural activities represents a key determinant of Indigenous Amazonian food security vulnerability [104]. This trend, however, also implies decreasing capacity to adapt to hazardous climatic events. Economic development may offer opportunities to improve local livelihoods, and therefore might increase adaptive capacity to face extreme climatic extreme events, yet lack of recognition of cultural values (e.g. food preferences and food systems activities) and nutritional importance of forest resources continues to exclude the Shawi from economic and nutritional benefits.

The predominance of non-climatic drivers influencing the short and long term adaptive capacity of Shawi food security under changing climatic conditions identified in this article suggest that the susceptibility of Shawi people to climate change is strongly related to cultural, socioeconomic and environmental changes rather than climatic risks per se. In addition, our findings about proximal and distal drivers of Shawi food insecurity, acting at a local levels but reinforced by past and current, socioeconomic governmental Peruvian policies, could help to explain why Indigenous Amazonian populations have consistently worse nutritional outcomes compared to non-Indigenous populations in the region.

A series of maladaptive trajectories have the potential to increase social and nutritional inequalities, and indicate that to assure a sustainable adaptive pathway for the Shawi population, transformational food security adaptation should include consideration of Indigenous perceptions and priorities, and should be part of Peruvian food and socioeconomic development policies.

## Supporting information

S1 File Instruments guidelines. (PDF)

S2 File Photovoice summary results. (PDF)
