# Supplementary material for: Multiple non-climatic drivers of food insecurity reinforce climate change maladaptation trajectories among Peruvian Indigenous Shawi in the Amazon
Source: PLoS One. 2018 Oct 16;13(10):e0205714. doi: 10.1371/journal.pone.0205714 (PMC6191111; doi:10.1371/journal.pone.0205714)
Supplement: S1 File — (PDF) [file pone.0205714.s001.pdf]

Interview guidelines

<table>
<tr><td colspan="2">Semi-structured interviews</td></tr>
<tr><td>Objective: To characterize practices for getting food throughout the year and how they are related to food availability, access and utilization</td><td>Participants: Key informants; Male and female, elderly</td></tr>
<tr><td colspan="2">General questions

Greetings, I would like to talk with you today about your community and about food. I am going to ask you about food in your community in the present, in the past and in the future. This interview will take approximately 45 minutes. Do you accept to talk with me?
1. Availability

What do you do to obtain your food? Is it the same throughout the year?

Do you share your food? Which foods?

Do you barter your food? What type of food and for what kind of goods?

Do you sell your food? How often? To whom? What do you usually buy with that money?

Do you have domestic animals like chickens, pigs or cows? How do you feed these animals?

Do you use domestic animals as food? Which animals? Under what circumstances?

Do you buy food? What food do you buy?
2. Access

During which part of the year do you eat well? What are factors that make this time a good time for getting food?

During which part of the year do you not eat well? What are factors that make this time a bad time for getting food? How do you deal with this?

What places are important for getting your food?

What kind of difficulties do you have to obtain your food?

What kind of food do you get in the community and what kind of food do you get in Yurimaguas?
3. Utilization

What food do you prefer?</td></tr>
</table>

What food do you consider is better for your health/ child/ pregnant women/elderly?

What food do you consider is worse for your health/ child/ pregnant women/elderly? Explain

How do you store and preserve your food?

How do you prepare your food?

4. Coping strategies

Do you remember a time when there was a severe scarcity of food for you and your family? Can you describe what happened?

What did you do to get food?

Do you remember a time when there was an abundance of food for you and your family? Can you describe what happened?

What would you do if someday your food production was damaged by drought/ flooding?

**Transect-walks**	
**Objective:** To explore crop production practices and constraints by accompanying participants to their lands	**Participants**: Key informants; Male and female, elderly

General questions

Greetings, I would like to talk with you today about your crops and farming practice. I would like to join you today to your land to better register your impressions and opinions. This interview will take approximately 45 minutes. Do you accept to talk and walk with me?

5. Time allocation

How many hours do you work in average?

Could you please describe, what kind of crops do you have in your land?

What is the most difficult time during the day for working your land? and why? Could you explain please?

What is the easiest time during the day for working your land? and why? Could you explain please?

Is there a time/month when you work in your land less hours than usual? and why? Could you explain please?

Is there a time/month when you work in your land more hours than usual? and why? Could you explain please?

In the past. Did people work the same than now in their land? and why? Could you explain please?

What are consequences of that change on your crop production?
