# Supplementary material for: Multiple non-climatic drivers of food insecurity reinforce climate change maladaptation trajectories among Peruvian Indigenous Shawi in the Amazon
Source: PLoS One. 2018 Oct 16;13(10):e0205714. doi: 10.1371/journal.pone.0205714 (PMC6191111; doi:10.1371/journal.pone.0205714)
Supplement: S2 File — (PDF) [file pone.0205714.s002.pdf]

Peruvian Indigenous Shawi in the Amazon talk about their food security
Photovoice results summary

"What helps you eat well? And what prevents you from getting food?
"¿Mata catawariken masha makatun kanan? ¿Uhpuaterata ku kusharo nanitere makakasu?"
"¿Qué te ayuda a alimentarte bien? Y ¿Qué te impide alimentarte bien?

- The objective of the Photovoice activity was to explore the food security experience, including the constraints and opportunities that would affect the Shawi's vulnerability to climate change.
- There were three groups of participants in this study: The first group included three male adolescents and four female adolescents aged 13 to 16 years old; the second group was comprised of 6 female adults ranging in age from 23 to 44 years old; and lastly, a male adult group was comprised of 4 people aged 28 to 37 years old.
- Two workshops were performed with each group. The first workshop aimed to explain what research means, to introduce the Photovoice methodology, to clarify the research question, to teach participants how to use digital cameras and to explain ethical aspects of the research. Ethics included individual and parental permission in the case of the minors, as well as recommendations regarding the privacy of people who potentially might appear in each photograph. Participants were instructed to avoid the inclusion of personal identifiers, such as faces, in the photographs in order to better preserve the privacy of community members.
- Although the original question for the Photovoice activity was: "What aspects of your everyday life affect what you eat and how much you have to eat?", after validation with the community and translation into Spanish and Shawi the question was modified for data collection to read: "What helps you eat well? And what prevents you from getting food? or, in Shawi, "¿Mata catawariken masha makatun kanan? ¿Uhpuaterata ku kusharo nanitere makakasu?"
- Each participant took photographs over the course of 2 to 3 days in order to answer this research question. During the second workshop, each group of participants was asked to select five to six photographs hat they considered to be most representative or important to answer the research question. These photographs were printed and then participants worked together to classify the pictures under themes that they considered to be important for their food security. Then, participants presented and discussed the organization of their final photographs under the different themes. Finally, each group of participants were asked to observe their selected photographs (pasted in cardboards) and to explain what they thought about the future of their food security. In the table below, we present some final photographs and themes from the Photovoice workshops, including the key quotes from participants in the group of adolescents, male adults, and female adults.

Photos were taken by Shawi male adolescents

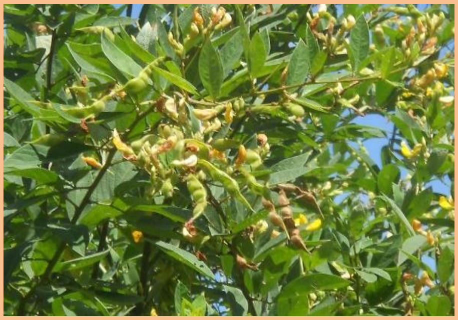

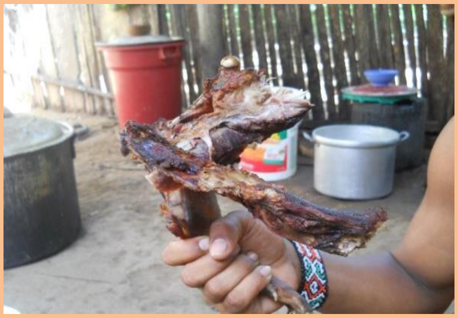

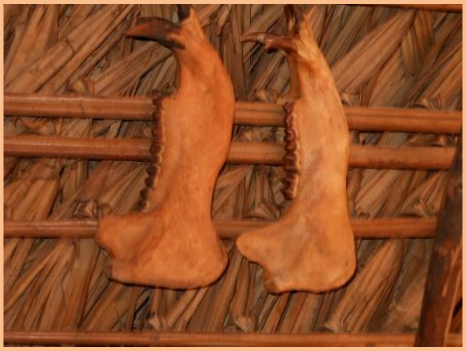

Theme: Foods that we eat in our daily lives
(Youth male participants)

"*We eat these nutrients in our country, Peru. We get these nutrients from our family garden. Sometimes you also go to the forest to look for something like deer, peccary.*"

"*These foods are eaten during the dry season and include fish and bushmeat*"

"*In our district, there is extreme poverty because people do not know how to harvest other products, but there are a few people who do know how. We would like to harvest rice, maize, cacao, sacha inchi, coffee, heart of palm and others. We would like to meet people who can teach us*"

Photos were taken by Shawi male adolescents	
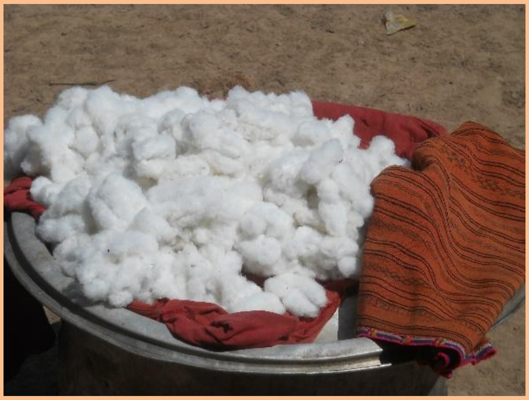 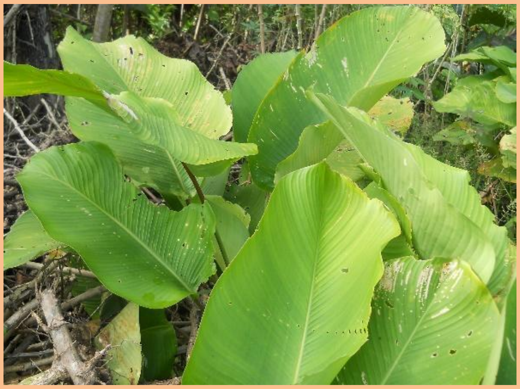 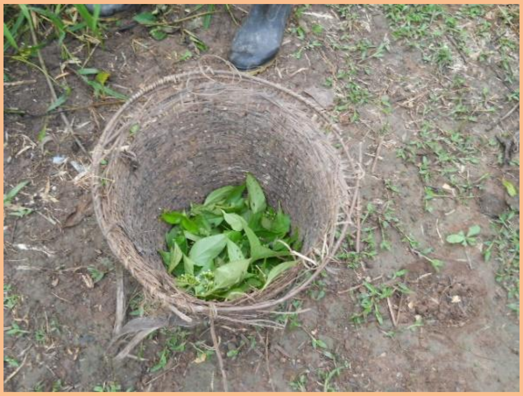	Theme: Plants that we need in order to bring food to our home (Youth male participants)<br><br>*"These are the plants that come from the mountain. The plants are for traditional medicine and also for the material women use to make skirts, similar to cotton"*<br><br>*"The leaves are using to wrap and prepare our food. There is also a plant call waca that is used to hunt fish in the river"*<br><br>*"There is a rope (tambishe) in the mountain that we use to make a basket called panero. We also bring wood from the forest for cooking at home; we get the wood from dry trees"*

Photos were taken by Shawi female adolescents

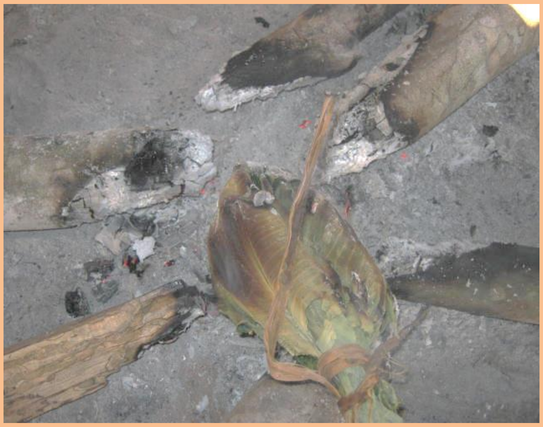

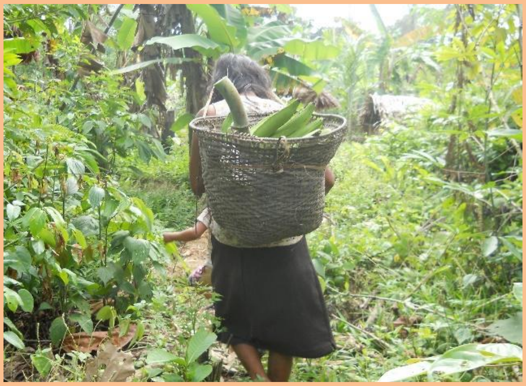

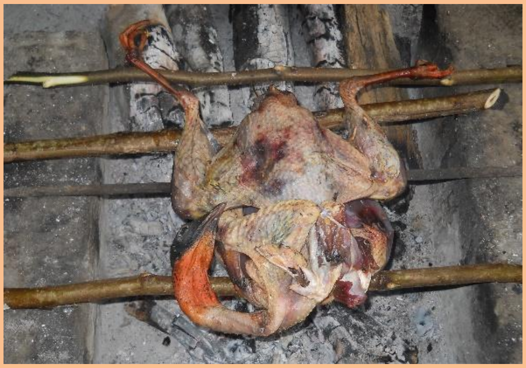

Theme: In our daily lives: Foods that we eat at home (Youth female participants)

"The men go to the mountain to get food. When they bring back fish, the women prepare meals. We use the leaves to prepare fish on the fire. When the food is ready, we serve it to the family and later we drink masato"

"The men take one to two days to hunt birds and meat like deer, peccary, perdiz, and agouti. Meanwhile, the mothers and daughters are harvesting plantain and getting wood so that it is ready when the father come back"

Photos were taken by Shawi female adolescents

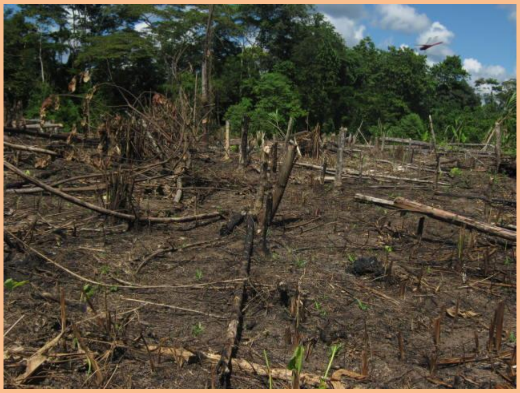

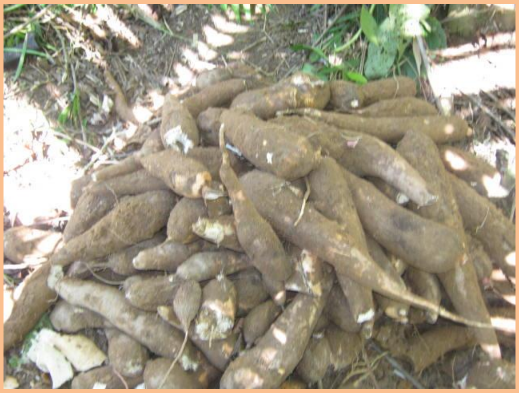

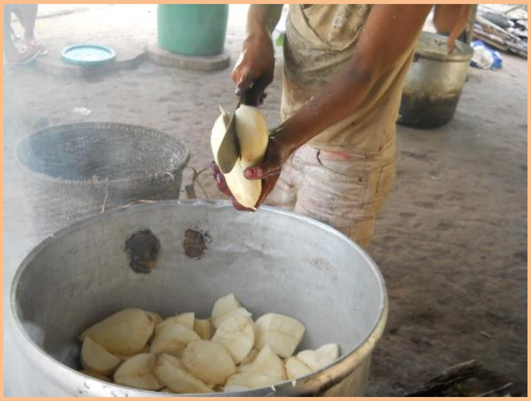

Theme: In our daily lives: Foods that we drink at home (Youth female participants)

"*To make a family garden, usually the men prepare the land by cleaning the forest and burning sticks. When the plot is ready, the whole family puts in cassava*"

"*Women take care of the cassava by weeding it everyday. Women harvest cassava, peel it, and put it in a basket (panero)*"

"*Cassava is washed in the river and then cooked in order to prepare masato*"

Group discussion - Shawi youth

When you look at these pictures, what do you think and what do you think about the future?

"*In the future, there will not be bushmeat. We probably will handle more money. We are going to be fine. We are going to have fewer children; in that way, we will be able to feed them better. We are going to keep our crops like cassava and plantain, of course only if we plant and take care of them*" (Male participant)

"*During the next few years, people will continue increasing in numbers. We are going to start to eat things that we never ate before like fox, dog, or cats. We are going to miss our fish, there be no animals in the forest, but the leaves in the forest will probably increase*" (Male participant)

"*I think I will have only one or two children. I will tell my partner that we do not have enough food to feed more children*" (Male participant)

"*We will continue doing our activities. We are going to continue preparing masato, taking care of our family gardens, and when there is no food, at least we are going to eat plantain*" (Female participant).

"*The cow probably will continue existing, sachapapa as well, but not the turtle or fish. If we have cassava, then we will have masato too. Cassava will not disappear because we plant a lot of it*" (Female participant)

"*I want to have only one or two children*" (Female participant)

"*If we were in the future, we will feel sad because we will not have meat, or patarashca (a fish based food). We are going to say, 'Twenty years ago we eat turtle and deer meat, but now we do not have that anymore' 'Before we had food, today there is no food*" (Female participant)

"*We are probably going to raise animals. When we look at these pictures, we also feel happy because there is food still for now*" (Female participant)

Photos were taken by Shawi male adults

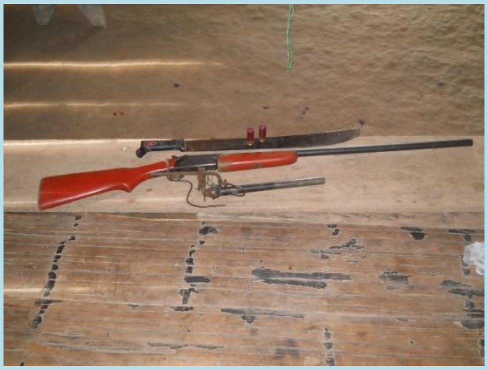

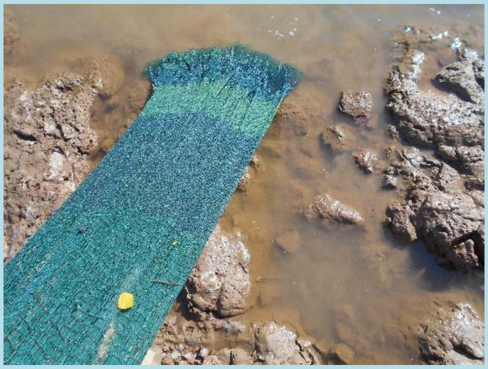

Theme: Tools for hunting animals from the forest
(Male adult participants)

“These tools are very important. Without them, a man is not a hunter”

“These tools are for getting game and fish from the forest”

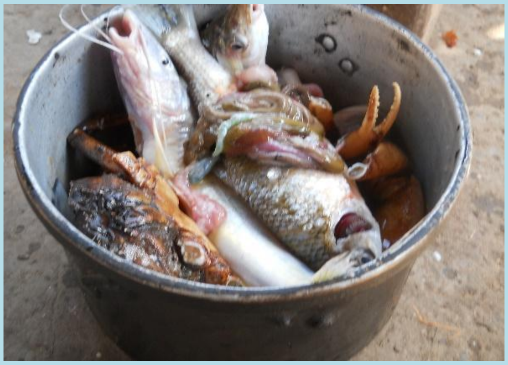

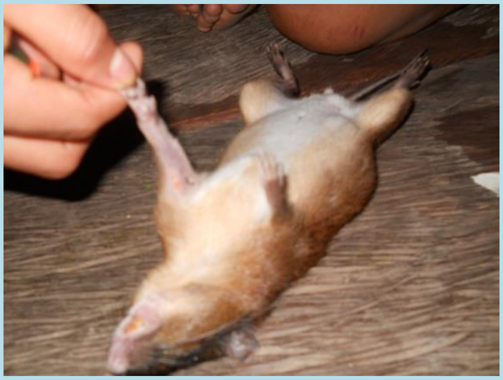

Theme: Food that is simple to get versus food that is difficult to get
(Male adult participants)

“*Those in this pictures, fish, small mammals and cassava are simple to get. However, there are others like deer, peccary, armadillo and monkeys that are more difficult because you need to walk long distances and it takes a long time to find them*”.

Photos were taken by Shawi male adults	
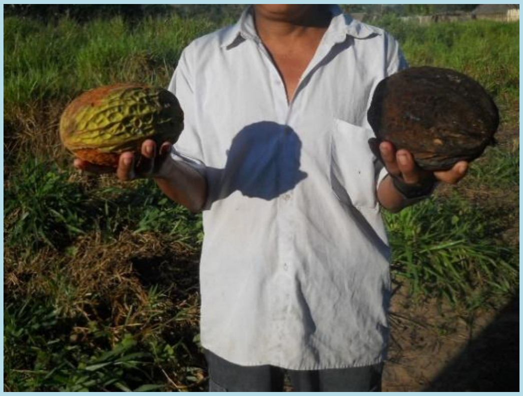 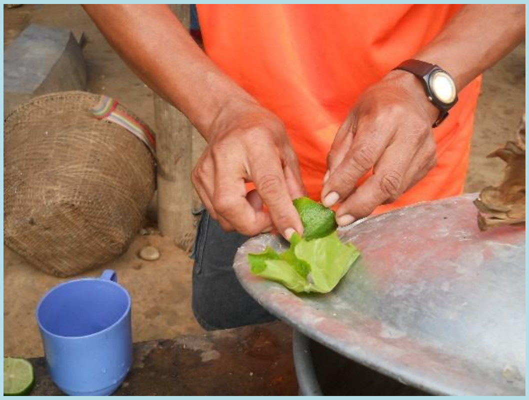	Theme: Key foods and conditions for getting food: Minor foods and health (Male adult participants)<br><br>“Necessary foods and the conditions that help us to get foods that are greater in size and quantity”<br><br>“Foods like *masato*, fruits, and plantain are important to eat everyday to stay well. Also, health is an important condition for procuring food”<br><br>“My father is in this picture making traditional medicine to cure my little brother’s diarrhea”

Group discussion-Shawi male adults

When you look at these pictures, what do you think and what do you think about the future?

"By observing tarrafa [fish net], we are sad because it is empty; there is no fish in it, same with the picture where the elder woman is eating only sachapapa. When you cannot get food, you don't bring anything to your home, and you get sad because you cannot feed your children" (Male adult participants)

"He is looking for fish to eat and then also looking for animals to hunt" (Male adult participants)

"When a child is sick, we are not going to leave him by himself. We are thinking about 'when my child will get better?', and we stay at home in case we need to take him to the health post. Only when the child gets better are you going to hunt" (Male adult participants)

By observing picture where there are fish, it makes you feel happy because they are going to eat today. Also the sachaycuy, because they are preparing to eat. Same with the macambo, because they will eat it" (Male adult participants)

"There are some food that will disappear, but others will continue existing. Different animals will no longer exist in the future include fish, sachacuy, Shiwi. We are going to have our tools, but without food to hunt". (Male adult participants)

"These food will continue: Cassava, masato, sachapapa, aguaje, water and macambo" (Male adult participants)

Photos were taken by Shawi female adults

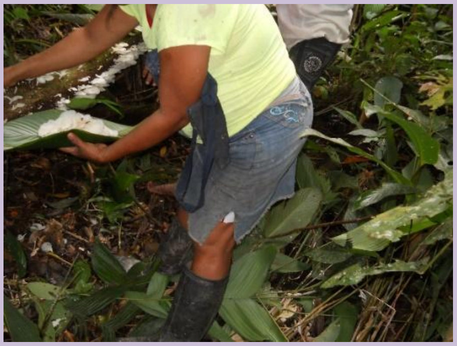

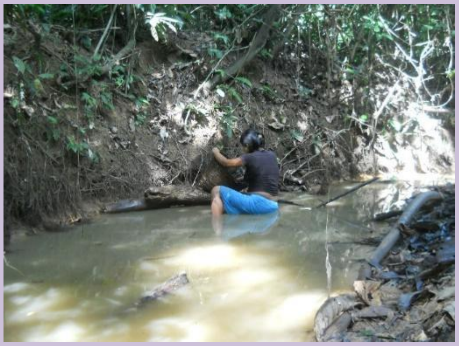

Theme: Activities for getting food

Sub-theme: in the forest/mountains
(Female adult participants)

"A woman is collecting wild fungus. You can get this all year round but mainly after two or three days of rain. Both men and women can do this activity".

"We are fishing in a stream. We are three women doing this activity. It is not a difficult activity, but it could be dangerous. We must be careful with river and terrestrial snakes"

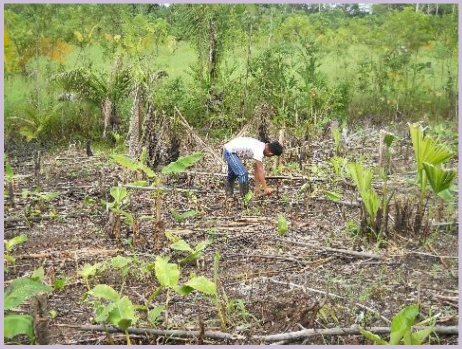

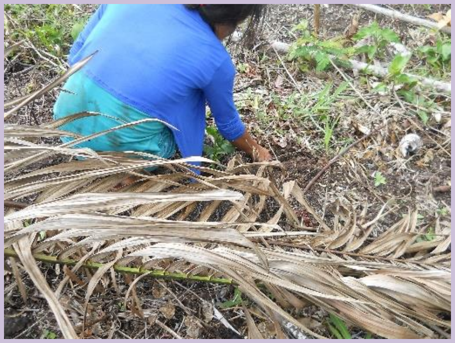

Sub-theme: Activities for getting food in the family gardens
(Female adult participants)

"This is a new plot of land. My son-in-law is making holes to put the cassava seeds into. He takes one week to make all the holes for planting cassava. My daughter is also working in here. We are both putting cassava in each hole. This is a hard activity. We cannot do this during wet season because you must burn bushes in order to prepare the land, and in the wet season you cannot make a fire well enough"

Photos were taken by Shawi female adults	
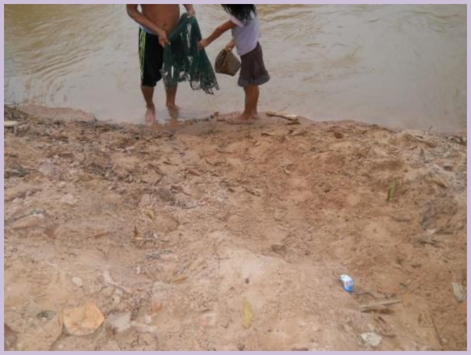<br>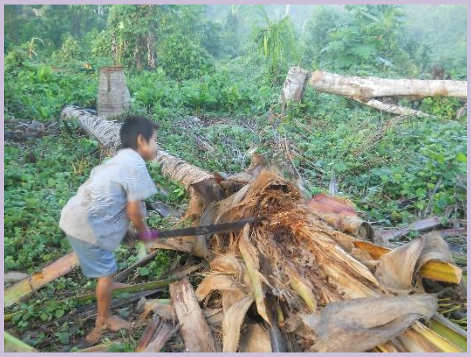	Sub-theme: Children get food<br>(Female adult participants)<br><br>*"Kids go look for food. Otherwise, whom will help them to get food?*<br>*"Hunger makes them look for food. Kids carry a traditional basket for fishing. Since no lunch is provided at school, after school, they can go to find fish using a fish hook"*<br><br>*"Kids look for food themselves because we are working in the minga [communal labor], or in the home garden, or in the forest looking for food ourselves"*<br><br>*"Kids can also find suri larva by searching inside palm trees"*

Photos were taken by Shawi female adults

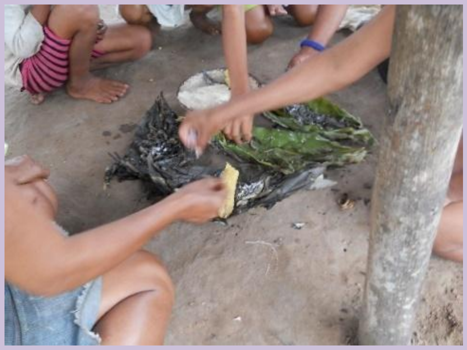

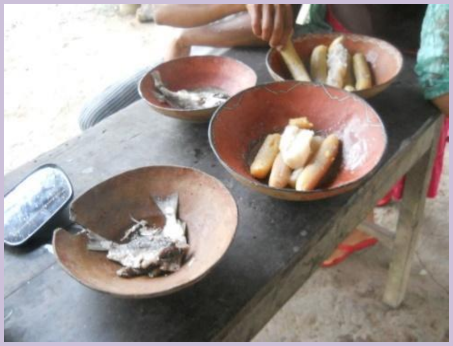

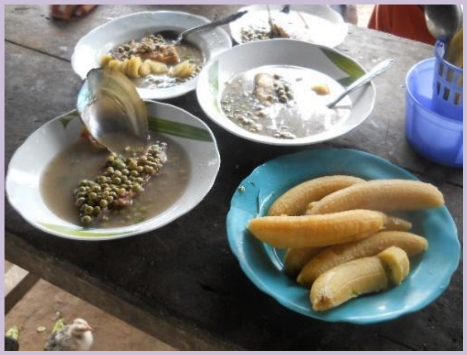

Theme: How we eat
(Female adult participants)

Sub-theme: As a group of relatives before going to the minga

“*We are eating small fish that the hostess family found in the forest. They have used the plant wuaca to catch this fish. This minga is to cut and clean the land to plant maize. Members of three households are participating in the minga*”

Sub-Theme: How we eat as a family

“We are eating fish. The first dish is for my husband, the second one is the plantain, the third dish is for me, my daughter, and my little grand daughter. When we have a bigger quantity of food, we separate the food onto different plates. When we only have a little food, we eat together. In this case we have a moderate amount of food”

“We have a type of local beans that are harvested only during the summer. We eat the beans with fish and plantain. We call this fish soup. These dishes are for my two children and me. We are using the Uri, which is a river shell that we use as spoon”

Photos were taken by Shawi female adults

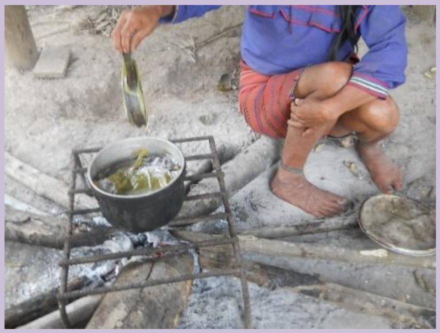

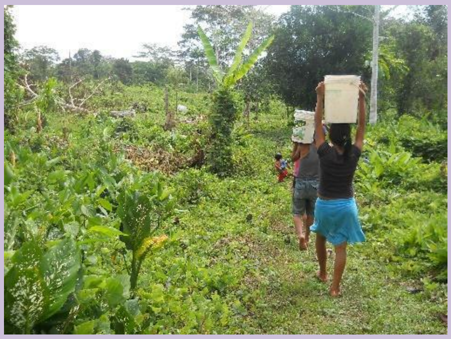

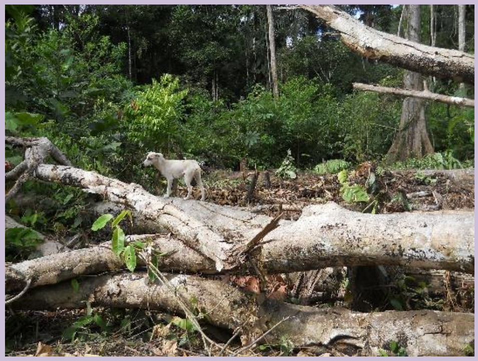

Theme: How we prepare food
(Female adult participants)

Sub-Theme: At breakfast

"She is preparing a fish called 'pango'. This is made with boiled plantain with some leaves on the top and fish on it. This is a normal food in the morning"

Sub-Theme: Getting water to prepare masato

"We use a basket to bring water from the closest river"

Sub-Theme: Getting firewood for cooking

"We also get firewood from family gardens. Wood is important. Otherwise, how am I going to cook my food? Usually my husband brings the firewood, but I or my children can get firewood too"

Sub-Theme: Female elders prepare food too

"The grandmother is serving masato to her granddaughter. She is 60 years old. Female elders can still cook. The grandmother brings cassava and prepares masato"

Group discussion-Shawi female adults

When you look at these pictures, what do you think and what do you think about the future?

(Female adult participants)

When we go fishing, we did not catch a lot. Then she comes back sad thinking 'And now, what will my child eat?'. Because of hunger, kids go to look for fish after school with their brother or little sister so they can have lunch" (Female adult participants)

"Drinking masato help us to feel better and be happy because when we go to work, we leave our children sad, so at least we have masato for drinking" (Female adult participants)

"When we are eating, we are happy. However, we feel sad because the fish will disappear. We think that in the future, we will have food from our gardens, but not from fishing" (Female adult participants)

"Looking at my family, I am thinking that at least their father is going to get a job to earn 10 soles so he can buy something to feed our children" (Female adult participants)
